# Reprogramming neuroblastoma by diet-enhanced polyamine depletion

**DOI:** 10.1038/s41586-025-09564-0

**Published:** 2025-09-24

**Authors:** Sarah Cherkaoui, Christina S. Turn, Yuan Yuan, Wenyun Lu, Lifeng Yang, Matthew J. McBride, Caroline Eigenmann, George E. Allen, Olesya O. Panasenko, Lu Zhang, Annette Vu, Kangning Liu, Yimei Li, Om H. Gandhi, Lea F. Surrey, Sandra D. Kienast, Sebastian A. Leidel, Michael Wierer, Eileen White, Joshua D. Rabinowitz, Michael D. Hogarty, Raphael J. Morscher

**Affiliations:** 1 Pediatric Cancer Metabolism Laboratory, Children’s Research Center, University of Zurich, Zurich, Switzerland; 2 Division of Oncology, University Children’s Hospital Zurich and Children’s Research Center, University of Zurich, Zurich, Switzerland; 3 Division of Oncology and Department of Pediatrics, Children’s Hospital of Philadelphia, Philadelphia, PA USA; 4 Perelman School of Medicine at the University of Pennsylvania, Philadelphia, PA USA; 5 Department of Health Sciences and Technology, Swiss Federal Institute of Technology (ETH Zürich), Zurich, Switzerland; 6 Department of Chemistry, Princeton University, Princeton, NJ USA; 7 Lewis-Sigler Institute of Integrative Genomics, Princeton University, Princeton, NJ USA; 8 Ludwig Institute for Cancer Research, Princeton Branch, Princeton University, Princeton, NJ USA; 9 Bioinformatics Support Platform, Faculty of Medicine, Geneva, Switzerland; 10 Department of Microbiology and Molecular Medicine, Institute of Genetics and Genomics Geneva, Faculty of Medicine, University of Geneva, Geneva, Switzerland; 11 BioCode: RNA to proteins (R2P) Platform, University of Geneva, Geneva, Switzerland; 12 Department of Molecular Biology and Biochemistry, Rutgers University, Piscataway, NJ USA; 13 Department of Molecular Biology and Biochemistry, Rutgers Cancer Institute of New Jersey, New Brunswick, NJ USA; 14 Department of Pathology and Laboratory Medicine, Children’s Hospital of Philadelphia, Philadelphia, PA USA; 15 Department of Chemistry, Biochemistry and Pharmaceutical Sciences, University of Bern, Bern, Switzerland; 16 Proteomics Research Infrastructure, Panum Institute, University of Copenhagen, Copenhagen, Denmark; 17 Division of Human Genetics, Medical University Innsbruck, Innsbruck, Austria

**Keywords:** Cancer metabolism, Paediatric research, Cell growth

## Abstract

Neuroblastoma is a highly lethal childhood tumour derived from differentiation-arrested neural crest cells^1,2^. Like all cancers, its growth is fuelled by metabolites obtained from either circulation or local biosynthesis^3,4^. Neuroblastomas depend on local polyamine biosynthesis, and the inhibitor difluoromethylornithine has shown clinical activity^5^. Here we show that such inhibition can be augmented by dietary restriction of upstream amino acid substrates, leading to disruption of oncogenic protein translation, tumour differentiation and profound survival gains in the *Th-MYCN* mouse model. Specifically, an arginine- and proline-free diet decreases the amount of the polyamine precursor ornithine and enhances tumour polyamine depletion by difluoromethylornithine. This polyamine depletion causes ribosome stalling, unexpectedly specifically at codons with adenosine in the third position. Such codons are selectively enriched in cell cycle genes and low in neuronal differentiation genes. Thus, impaired translation of these codons, induced by combined dietary and pharmacological intervention, favours a pro-differentiation proteome. These results suggest that the genes of specific cellular programmes have evolved hallmark codon usage preferences that enable coherent translational rewiring in response to metabolic stresses, and that this process can be targeted to activate differentiation of paediatric cancers.

## Abstract (web summary)

Enhanced polyamine depletion in neuroblastoma models decreases translation of mRNA codons with adenosine in the third position, reprogramming the tumour proteome away from cell cycle progression and towards differentiation.

## Main

Hyperactive MYC signalling via amplification of the *MYCN* proto-oncogene is a hallmark of neuroblastoma^6^ and drives aggressive disease with poor outcomes^7–9^. In the *Th-MYCN* genetically engineered mouse model, enforced *MYCN* expression in sympathoadrenal cells induces neuroblastomas^10^. The rate-limiting enzyme in polyamine biosynthesis, ornithine decarboxylase (ODC), is directly transcriptionally upregulated by MYCN^11^ and is often co-amplified^12^. Inhibition of ODC by difluoromethylornithine (DFMO) has recently been approved by the US Food and Drug Administration for treatment of children with high-risk neuroblastoma^5^. Combined treatment strategies to enhance DFMO activity are therefore of great interest.

One potential such strategy is depletion of the ODC substrate ornithine. Ornithine can be derived from arginine via a single enzymatic step catalysed by arginase, or from proline and glutamine via two or three steps that converge on the enzyme ornithine aminotransferase (OAT)^13^. In early childhood, most ornithine comes from proline via OAT^14,15^. Since neuroblastomas arise in early childhood, they might similarly depend on OAT, which was recently implicated as a metabolic driver in pancreatic cancer through the provision of ornithine and thereby polyamines^16^.

## Altered metabolism in MYCN neuroblastoma

**Fig. 1:**
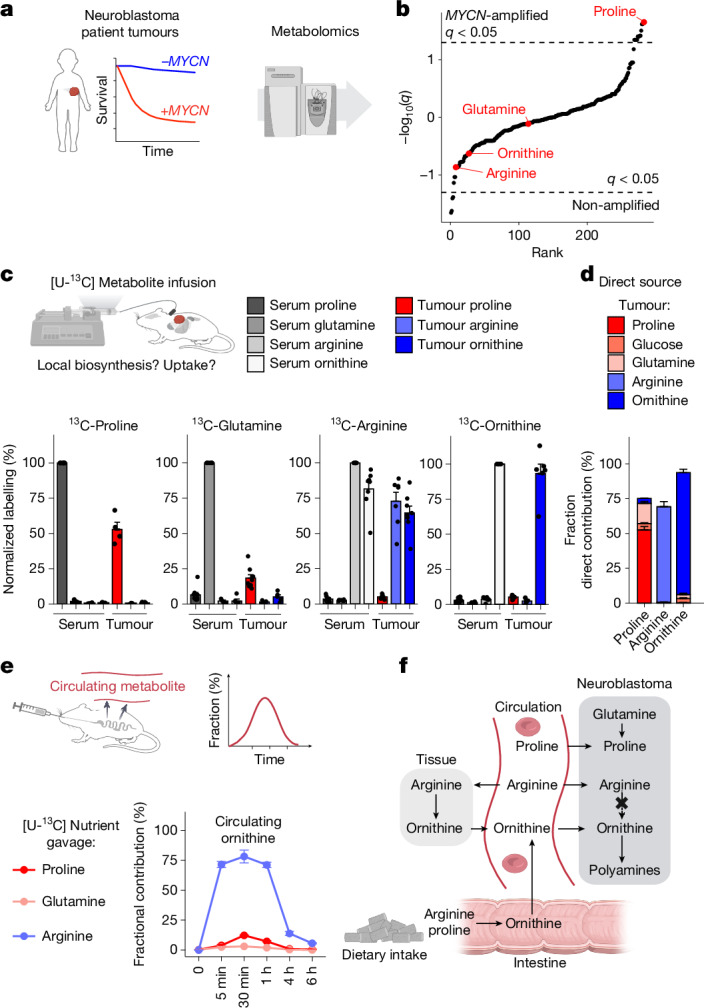
*MYCN*-driven neuroblastoma tumours are characterized by high proline levels and a functionally disconnected proline and arginine metabolism that is dependent on uptake from circulation. **a**, Primary neuroblastoma tumour tissue was analysed using liquid chromatography–mass spectrometry-based metabolomics. **b**, Differential abundance of 303 metabolites. Proline was the most significantly increased metabolite in *MYCN-*amplified primary human neuroblastoma relative to non-amplified tumours. Dotted line marks the significance threshold, with *P* values corrected for a false discovery rate (FDR) of 0.05 (*q* < 0.05; *n* = 10). **c**, In vivo stable isotope tracing identifies the circulating precursors of intratumoral metabolites. Labelling is normalized to the serum for each infused [U-^13^C] metabolite in *Th-MYCN* mice fed a chow diet. Data are mean ± s.e.m. Proline serum: *n* = 6; proline tumour: *n* = 4; glutamine serum: *n* = 9; glutamine tumour: *n* = 9; arginine serum: *n* = 8; arginine tumour: *n* = 8; ornithine serum: *n* = 7; ornithine tumour: *n* = 6. **d**, Direct circulating nutrient contributions to tumour tissue metabolite pools of proline, arginine and ornithine in *Th-MYCN* mice. The colour indicates the respective circulating nutrient source. Contributions derived from [U-^13^C]-labelled tracer infusions, derived from data shown in **c**. Data are mean ± s.e.m. **e**, Oral gavage of ^13^C-labelled nutrients shows the dietary contribution to circulating ornithine. The gavage feed introduces one-third of the daily intake of the respective amino acid in its [U-^13^C] form, which is used to quantify its contribution to polyamine-related downstream metabolites over time. Feeds in the *Th-MYCN* model are adapted to mouse weight. Data are mean ± s.e.m., *n* = 6. **f**, Schematic of tumour metabolite sources in neuroblastoma. The amino acids proline and arginine are primarily taken up from circulation. Tracing identifies the polyamine precursor ornithine to be primarily derived from circulation and not from intratumoral biosynthesis. Arginine is the primary circulatory substrate for ornithine production. In the intestine, ornithine is produced from arginine and proline through OAT activity. Panels **a**, **c**, **e** and **f** created in BioRender. Morscher, R. (2025) https://BioRender.com/ntr5665 (**a**); https://BioRender.com/x0bpqyh (**c**); https://BioRender.com/50lb1xh (**e**); https://BioRender.com/3h88n1g (**f**). Source data

**Extended Data Fig. 1:**
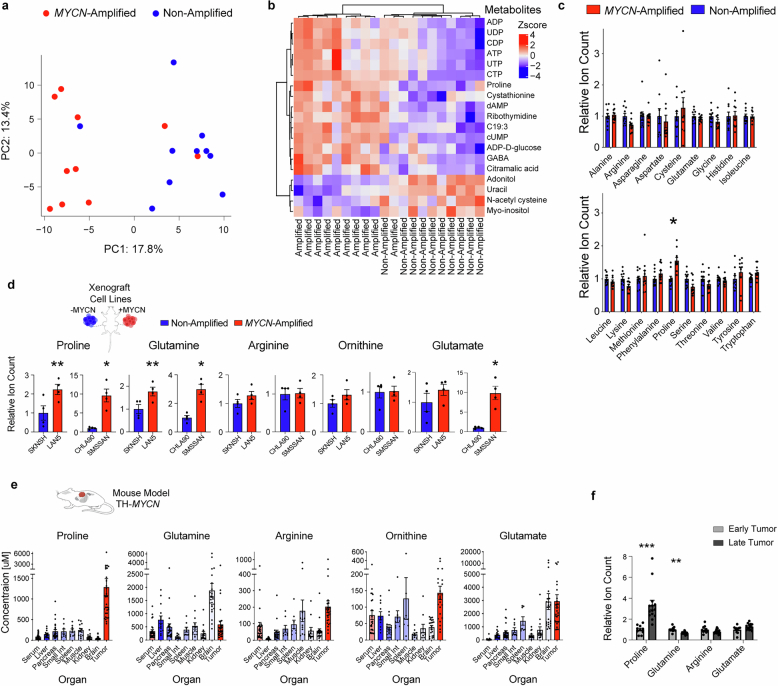
Metabolomic profiling of *MYCN*-amplified primary patient tumors and xenografts reveals reprogramming of the arginine-proline-glutamine axis. a) Global metabolomic signatures of primary human neuroblastoma tumors analyzed by principal component analysis, with *MYCN-*amplified (red) and non-amplified (blue). b) Heatmap of significantly changed metabolites in primary neuroblastoma tumor samples (q < 0.05), comparing *MYCN-*amplified to non-amplified tumors. Unsupervised clustering performed using Ward’s method. c) Levels of all proteinogenic amino acids in primary neuroblastoma tumor tissue. Data in a-c as in Fig. 1b with *n* = 10 each group and p-values corrected for false discovery rate.*q < 0.05. Mean ± s.e.m. d) Relative levels of polyamine related metabolites in bilateral xenografts from respective *MYCN-*amplified and non-amplified neuroblastoma cell lines. *P < 0.05, **P < 0.01, two-tailed paired t-test. Mean ± s.e.m., *n* = 4. e) Proline concentration in *MYCN*-driven neuroblastoma tumors is significantly increased in the *Th*-*MYCN* mouse model, whereas glutamine, arginine, ornithine and glutamate levels across organs are within physiological range. **d**,**e**, Mean ± s.e.m., proline tumor *n* = 31 other organs *n* = 8-31, glutamine tumor *n* = 29 other organs *n* = 8-29, arginine tumor *n* = 25 other organs *n* = 8-25 and tumor ornithine *n* = 24; glutamate tumor *n* = 23 other organs *n* = 8-25. f) Relative metabolite levels across tumors harvested at early (< 50 mm^3^) compared to the late timepoint in the *Th*-*MYCN* model. **P < 0.01, ***P < 0.001, Two-tailed t-test. Mean ± s.e.m., early *n* = 10, late *n* = 14. Panels d and e created in BioRender. Noureddine, N. (2025) https://BioRender.com/vxtxvhq. Source data

**Extended Data Fig. 2:**
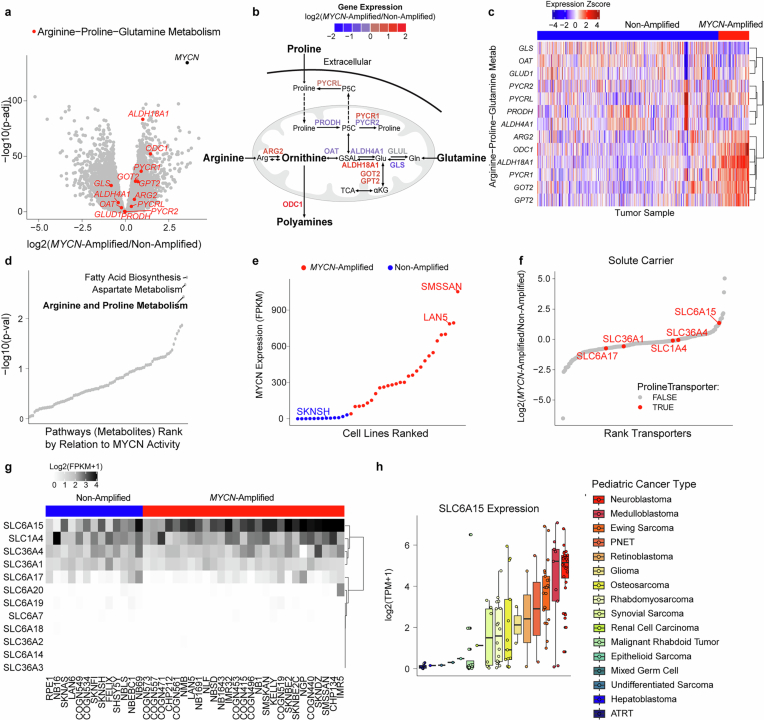
Arginine and proline metabolism expression in primary neuroblastoma patients and cell lines. a) Differential gene expression between *MYCN*-amplified and non-amplified primary human neuroblastomas. Genes related to arginine, proline and glutamine metabolism are denoted in red, *MYCN* in black and all other genes in grey. b) More detailed schematic of gene expression levels of enzymes across arginine, proline and glutamine metabolism with the color of each gene label indicating relative expression (*MYCN-*amplified/non-amplified). c) Heatmap of gene expression from genes related to arginine, proline and glutamine metabolism according to MYCN status. d) *MYCN* transcriptional signatures from gene expression associate with metabolite levels of the arginine and proline metabolic pathways. Data from metabolome profiling of *n* = 180 cancer cell lines. e) *MYCN* expression across neuroblastoma cell lines, colored by *MYCN* amplification status. Cell lines selected for xenografts are depicted by their name (Extended Data Fig. 1d). CHLA90, not in this dataset, is a *MYCN* non-amplified line. f) Relative expression of solute carrier group members (SLC) according to *MYCN* amplification status across neuroblastoma cell lines from Harenza et al. Proline transporters are highlighted in red. g) Proline transporter expression identifies SLC6A15 as the primary proline transporter and its upregulation in relation to *MYCN* amplification status in neuroblastoma cell lines. h) Expression of the proline transporter SLC6A15 across paediatric cancer cell lines highlights its expression across paediatric cancers. The center line represents the median, the box spans the interquartile range (IQR; 25th to 75th percentiles), and whiskers extend to 1.5I QR. Data points beyond this range are shown as outliers (solid black). *n* = 1-28 per cancer. Graphs a-c display primary neuroblastoma expression data. *MYCN-*amplified *n* = 93, Non-amplified *n* = 551. Graphs e, f and g display neuroblastoma cell line expression data. *MYCN-*amplified *n* = 27, Non-amplified *n* = 13. Source data

We found that proline, whose catabolism can feed into ornithine via OAT, is strongly increased in MYCN-driven neuroblastoma. This was shown in three contexts: primary patient tumours exhibiting *MYCN* amplification (relative to unamplified tumours), xenografts induced from patient-derived neuroblastoma cell lines exhibiting high *MYCN* expression (relative to cell lines with low *MYCN* expression), and *Th-MYCN* genetically engineered mouse tumours (relative to normal organs of the mouse) (Fig. 1a,b, Extended Data Fig. 1 and Supplementary Table 1). In the *Th-MYCN* model, proline content was also markedly higher in late tumours (those larger than 50 mm^3^) compared with early tumours (Extended Data Fig. 1f). Gene-expression analysis indicated that proline transport and de novo biosynthesis is upregulated in *MYCN*-amplified patient tumours and cell lines (Extended Data Fig. 2), which is of interest as recent work has highlighted the necessity of reductive proline biosynthesis^17^. Levels of ornithine and other upstream precursors were not consistently higher in these cells. Thus, we identified a large increase in proline in MYCN-driven neuroblastoma and propose proline as a candidate target for potential enhancement of neuroblastoma therapy.

**Extended Data Fig. 3:**
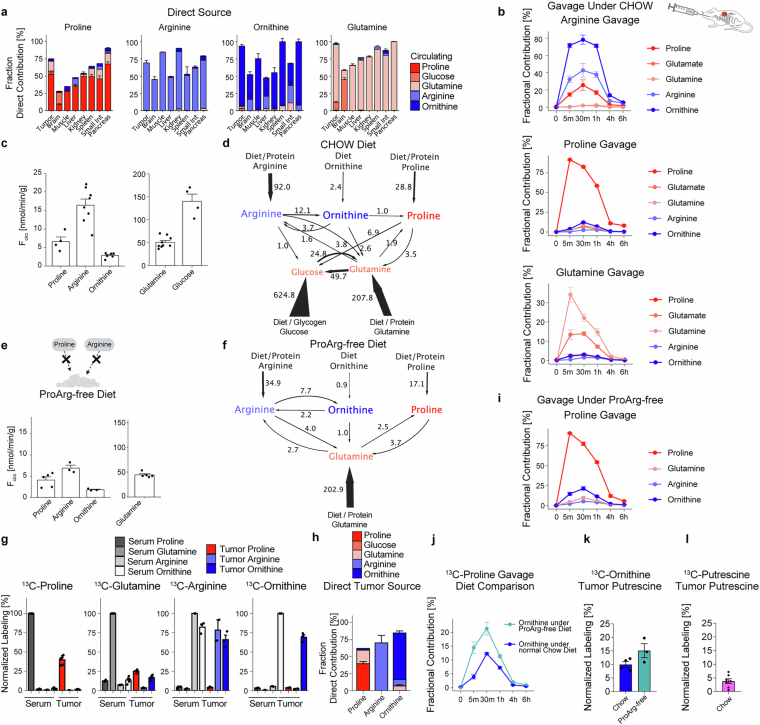
Direct metabolite contributions across organs and turnover flux in the* Th*-*MYCN* model. a) Direct contributions of circulating nutrients to proline, arginine, ornithine and glutamine across organs and neuroblastoma tumors under a normal chow diet. Contributions derived from [U-^13^C]-labelled tracer infusions as given in the legend, complementing Fig. 1c. Mean ± s.e.m. b) Oral application of labeled nutrients identifies the dietary precursors of circulating metabolites. Gavage with [U-^13^C]arginine, proline and glutamine at a dose of 1/3 of a daily consumption of the respective amino acids. The graph indicates fractional labeling in the serum of related polyamine related metabolites over time in mice fed a normal chow diet. Mean ± s.e.m., *n* = 6. c) Circulatory serum turnover flux, F_circ_ of proline, arginine, ornithine, glutamine and glucose under normal chow. Mean ± s.e.m., proline *n* = 4, arginine *n* = 8, ornithine *n* = 6, glutamine *n* = 9, glucose *n* = 4. d) Whole-body flux model of interconversion between sources of circulating proline, ornithine, arginine, glutamine and glucose in nmol C/min/g under normal chow. Mean fluxes above 1 nmol C/min/g are displayed. e) Circulatory serum turnover flux under a proline and arginine-free diet, F_circ_ of proline, arginine, ornithine and glutamine. Mean ± s.e.m., proline *n* = 5, arginine *n* = 3, ornithine *n* = 3, glutamine *n* = 5. f) Whole-body flux model of interconversion between sources of circulating proline, ornithine, arginine and glutamine in nmol C/min/g under a ProArg-free diet. Mean fluxes above 1 nmol C/min/g are displayed. g) In vivo stable isotope tracing-based assessment of circulating precursors to intratumoral metabolites in *Th*-*MYCN* mice fed a ProArg-free diet. Labeling is given normalized to the serum of each infused [U-^13^C]metabolite. Mean ± s.e.m. Tracing: Proline serum *n* = 5, tumor *n* = 5; Glutamine serum *n* = 4, tumor *n* = 4; Arginine serum *n* = 3, tumor *n* = 3; Ornithine serum *n* = 3, tumor *n* = 3. h) Direct contributions of circulating nutrients in neuroblastoma tumors under a ProArg-free diet. Contributions derived from [U-^13^C]-labelled tracer infusions complementing Fig. 1h. Mean ± s.e.m., serum and tumor. i) Oral gavage of stable isotope tracing elucidates the dietary precursors of circulating metabolites. Gavage with [U-^13^C]proline bolus and fraction contribution labeling in the serum of related amino acids and polyamine precursor ornithine in mice fed a ProArg-free diet prior. Mean ± s.e.m., *n* = 6. j) Intestinal proline to ornithine conversion doubles in mice fed a ProArg-free diet, reaching more than 20% serum ornithine enrichment after a [U-^13^C]proline feed. A ProArg-free diet is used to minimize non-labelled proline flux from interconversion to ornithine. Mean ± s.e.m., *n* = 6. k) Putrescine labeling in tumor indicates contribution from ornithine infusion after 5 h. Normalized labeling from infusion under a normal chow and ProArg-free diet. Mean ± s.e.m., Chow *n* = 4; ProArg-free *n* = 3. l) Putrescine labeling in tumor indicates contribution from putrescine infusion after 5 h. Normalized labeling from infusion under a normal chow diet. Mean ± s.e.m. *n* = 7. Abbreviation: Small int., small intestine; ProArg-free, proline and arginine free. Panel b created in BioRender. Morscher, R. (2025) https://BioRender.com/5p7gqdc. Source data

As a complementary approach to identify metabolic targets, we first investigated the in vivo circulatory sources of proline and ornithine using infusion-based stable isotope tracing in the *Th-MYCN* model^18–20^. Tumour proline was derived from circulating proline and glutamine, reflecting proline acquisition from a combination of circulatory uptake and de novo biosynthesis (Fig. 1c). Unlike in previous studies in infants^21,22^, this proline was not converted to ornithine in substantial quantities in the neuroblastoma tumours. Examination of gene-expression data from primary tumours revealed that *OAT* expression is low in MYCN-driven neuroblastoma (Extended Data Fig. 2b), consistent with the need for an alternative ornithine source rather than intratumour synthesis from glutamine or proline. Isotope tracing confirmed the source to be circulating arginine and ornithine itself. Most circulating ornithine was derived from arginine (Fig. 1c and Extended Data Fig. 3) and to a lesser extent from dietary, but not circulating, proline (Fig. 1e). A large fraction of oral arginine was also converted to circulating proline, highlighting carbons being diverted from ornithine to proline synthesis by intestinal OAT activity (Extended Data Fig. 3b). Tumour ornithine was most strongly labelled from circulating ornithine, although it was also strongly labelled from circulating arginine. Quantitative modelling analysis of tumour ornithine sources revealed that circulating arginine feeds tumour ornithine mainly indirectly, after being converted to ornithine elsewhere in the body, and subsequent uptake of the resulting circulating ornithine by the tumour (Fig. 1d and Extended Data Fig. 3c,d). The ultimate upstream source of most neuroblastoma ornithine under a standard diet is therefore arginine, highlighting arginine restriction as a potential complement to proline restriction and DFMO treatment for neuroblastoma therapy.

To test this, we introduced a proline- and arginine-free (ProArg-free) diet (Supplementary Table 2) in the *Th-MYCN* mouse model and assessed its effect on metabolic networks by in vivo tracing. The ProArg-free diet reduced circulating fluxes of proline, arginine, glutamine and ornithine (Extended Data Fig. 3e,f). Nonetheless, infusion-based tracing showed largely unchanged labelling of intratumoral polyamine-related metabolites, with uptake from circulation remaining the predominant source of ornithine (Extended Data Fig. 3g). This indicates that even under a diet depleted of its ornithine substrates, intratumoral OAT directionality is maintained with only minor local ornithine production in neuroblastoma (Extended Data Fig. 3f,h). By contrast, pre-circulatory intestinal conversion of dietary proline to ornithine via OAT doubled under the ProArg-free diet to support systemic ornithine levels (Extended Data Fig. 3i,j). Ornithine is then converted to polyamines locally in the tumours, as evidenced by the contribution to putrescine labelling, with increased labelling from ornithine upon a ProArg-free diet (Extended Data Fig. 3k) and lower contribution from circulating putrescine (Extended Data Fig. 3l). We therefore evaluated the anti-tumour effect of dietary substrate depletion of the two major amino acid precursors of ornithine (Fig. 1f) in combination with DFMO treatment.

## Combining a ProArg-free diet with DFMO

**Fig. 2:**
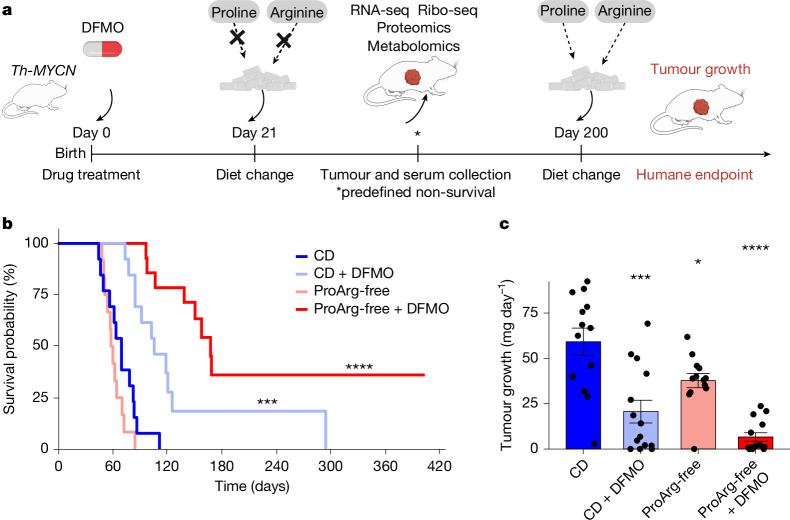
A ProArg-free diet enhances tumour growth suppression by DFMO in *MYCN*-driven neuroblastoma. **a**, Schematic of two-factor intervention, including the ProArg-free diet (from day 21) and DFMO treatment via the drinking water (1%, from day 0 to nursing mothers and directly to pups from day 28) in the *Th-MYCN* genetically modified mouse model. **b**, Kaplan–Meier curve of tumour-free survival with combined CD or ProArg-free diet plus DFMO. *P* value from log-rank test compared to CD. **c**, Tumour growth, defined as tumour mass at death normalized by day of life. Two-tailed *t*-test compared to CD. Data are mean ± s.e.m. CD: *n* = 13; CD + DFMO: *n* = 14; ProArg-free: *n* = 13; ProArg-free + DFMO: *n* = 14. *P < 0.05, **P < 0.01, ***P < 0.001, ****P < 0.0001. Panel **a** created in BioRender. Morscher, R. (2025) https://BioRender.com/n9dgse0. Source data

Using the *Th-MYCN* model, we next examined the effect of combined dietary amino acid depletion (proline and/or arginine) with or without pharmacological ODC inhibition by DFMO (Fig. 2a). Mice fed the ProArg-free diet alone showed reduced neuroblastoma growth compared with control diet (CD), with no effect on tumour-free survival (Fig. 2b,c). As in previous studies, inhibition of polyamine biosynthesis by DFMO monotherapy extended survival^23–25^. In mice with prolonged survival, lethal tumour progression was observed after treatment cessation.

**Extended Data Fig. 4:**
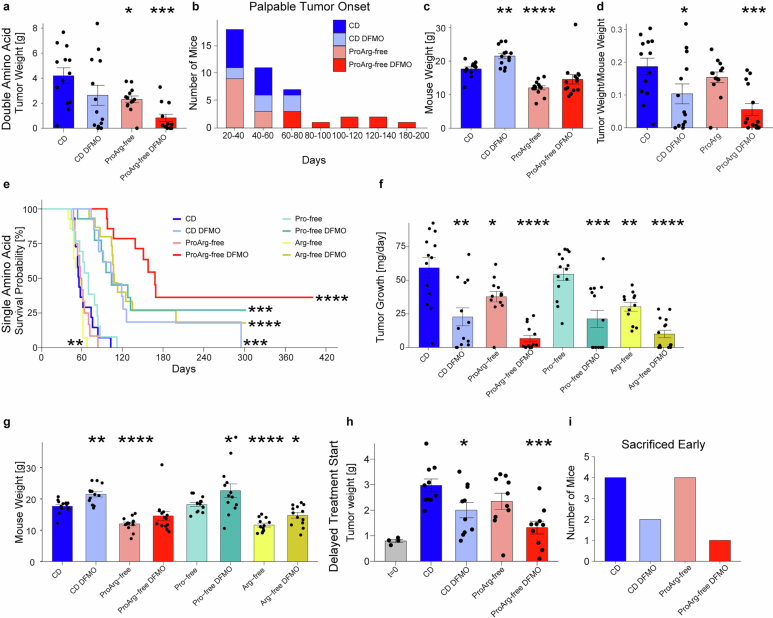
Effect of proline and arginine amino acid depletion combined with DFMO treatment on *MYCN*-driven neuroblastoma in vivo. a) Tumor weight at death in grams for the respective treatment groups. b) Delayed tumor growth as shown by time to palpable tumor onset across all treatment arms. c) Mouse weight at death in grams, adjusted for tumor weight.d) Tumor weight at death in grams, adjusted for mouse weight. e) Kaplan-Meier curve of tumor-free survival in the *Th*-*MYCN* model. Treatment groups undergo diet change at day 21 and DFMO treatment via drinking from day 0 and encompass either diet change alone (CD, Pro-free, Arg-free and ProArg-free) or their combination with DFMO. Related to Fig. 2, single amino acid depletion added. f) Tumor growth defined as tumor weight at death normalized by day of life in the single amino acid trial. Mean ± s.e.m. g) Mouse weight under arginine or proline single amino acid free diets compared to combined ProArg-free diets (also in c). h) Tumor weight at sacrifice after late-stage treatment start in the *Th*-*MYCN* model (palpable abdominal tumors). Treatment was limited to 14 days after which the mice were sacrificed to obtain tumor weight as primary endpoint. i) Number of mice that were sacrificed before endpoint. In the CD treatment group, four mice died before reaching 14 days, compared to only 1 in ProArg-free-DFMO group. For a, c, d, f g and h: two-tailed t-test compared to CD. *P < 0.05, **P < 0.01, ***P < 0.001, ****P < 0.0001. 0.0007. Mean ± s.e.m. For e: Log-rank test p-value compared to CD. For a-g *Th*-*MYCN* model, related to Fig. 2: CD *n* = 13, CD DFMO *n* = 14, ProArg-free *n* = 13, ProArg-free DFMO *n* = 14, Arg-free *n* = 14, Arg-free *n* = 15, Pro-free *n* = 15, Pro-free DFMO *n* = 15. For h-i Delayed treatment *Th*-*MYCN* model: CD *n* = 11, CD DFMO *n* = 10, ProArg-free *n* = 10, ProArg-free DFMO *n* = 9. Abbreviations: CD treatment, control diet; Pro, proline; Arg, arginine; Free, diet free; DFMO, difluoromethylornithine. Source data

Notably, combining dietary proline and arginine removal with DFMO induced a marked survival benefit (Fig. 2b), decreased tumour growth (Fig. 2c) and increased time to detectable tumour (Extended Data Fig. 4a,b). One-third of mice in the ProArg-free diet plus DFMO regimen had extended survival, with approximately 20% remaining tumour-free, as confirmed via necropsy. Although the ProArg-free diet caused a reduction in mouse weight, this did not affect survival and was not worsened by adding DFMO (Extended Data Fig. 4c,d). The therapeutic effect of combining DFMO with dietary depletion of either proline or arginine alone was inferior to DFMO plus ProArg-free diet (Extended Data Fig. 4e–g). Even when delaying treatment initiation until pre-terminal tumour progression, we observed a significant reduction in tumour mass in the ProArg-free diet plus DFMO regimen, suggesting relevance for treatment of established tumours (Extended Data Fig. 4h,i). Despite the unfavourable metabolic environment, a higher immune cell infiltration and stromal component was observed in the ProArg-free, DFMO-treated tumours (Supplementary Fig. 1). In summary, the ODC inhibitor DFMO, which was recently approved by the US Food and Drug Administration for neuroblastoma treatment, combined with a diet free of the non-essential amino acids arginine and proline, significantly augments anti-tumour activity with around 20% of treated mice remaining tumour-free 100 days beyond the end of therapy.

## ProArg-free diet plus DFMO enhances polyamine depletion

**Fig. 3:**
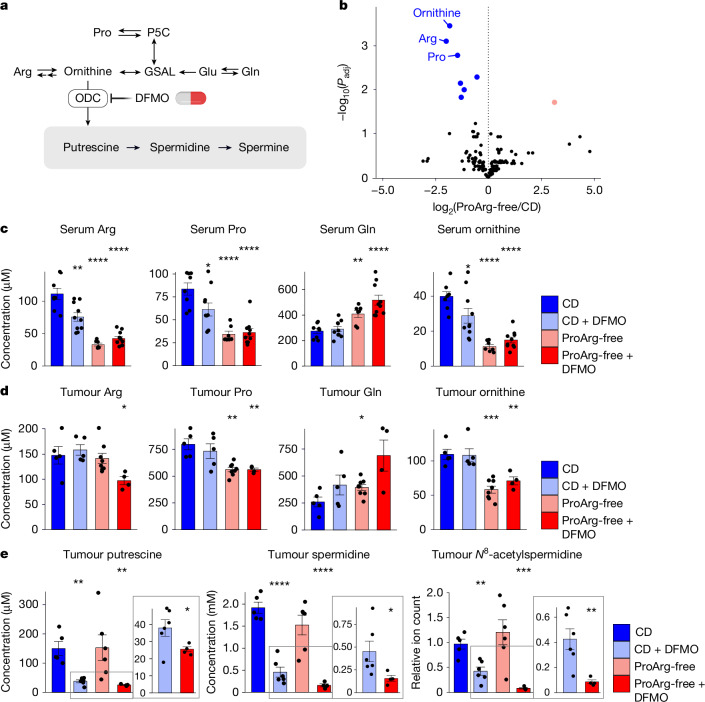
Dietary intervention causes substrate depletion to enhance polyamine biosynthesis inhibition by DFMO. **a**, Schematic of arginine, proline and glutamine metabolism and its direct link to polyamines via ornithine. GSAL, glutamate-γ-semialdehyde; P5C, pyrroline-5-carboxylate. **b**, Differential serum metabolite levels comparing ProArg-free diet with CD. Blue dots highlight metabolites that are significantly depleted (FDR < 0.05) and the rose dot indicates a metabolite that was upregulated compared with CD. CD: *n* = 8; ProArg-free: *n* = 7. **c**, Serum arginine, proline, glutamine and ornithine across groups. Statistical comparisons to CD. Data are mean ± s.e.m. CD: *n* = 8; CD + DMFO: *n* = 10; ProArg-free: *n* = 7; ProArg-free + DFMO: *n* = 7. **d**, Tumour arginine, proline, glutamine and ornithine levels reveal dysregulation of arginine and proline metabolism with combined ProArg-free diet plus DFMO treatment. Average age at end point is eight weeks. Statistical comparisons to CD. Data are mean ± s.e.m. CD: *n* = 5; CD + DMFO: *n* = 5; ProArg-free: *n* = 8; ProArg-free + DFMO: *n* = 4. **e**, A ProArg-free diet enhances polyamine depletion in tumour tissue induced by DFMO in prolonged treatment. Average age at end point is 12 weeks. Statistical comparisons to CD. Insets, magnified graphs highlight the additional difference in polyamine levels induced by ProArg-free diet over DFMO only. Data are mean ± s.e.m. CD: *n* = 5; CD + DMFO: *n* = 5; ProArg-free: *n* = 6; ProArg-free + DFMO: *n* = 4. Two-tailed *t*-test. *n* denotes the number of mice measured by metabolomics. Source data

**Extended Data Fig. 5:**
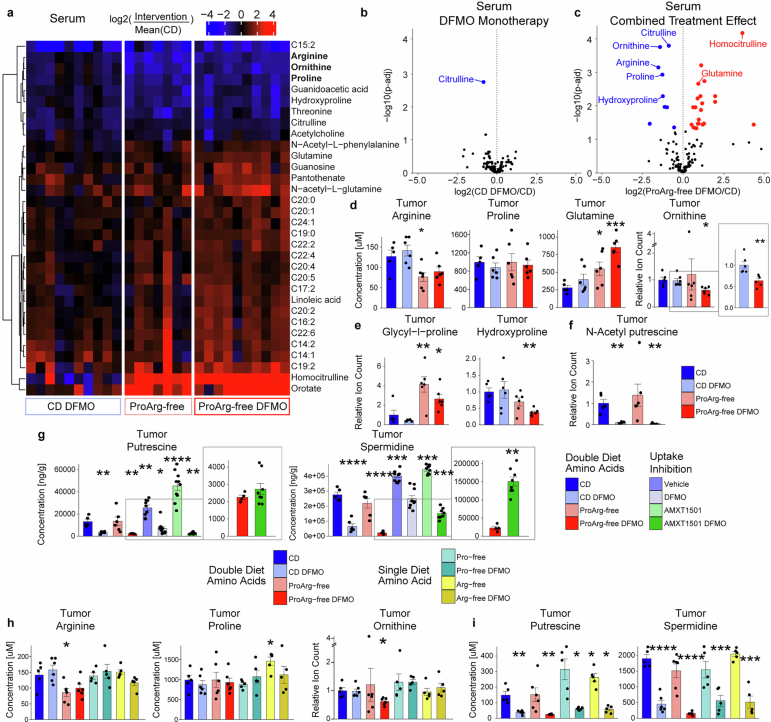
Metabolite profiles of serum and tumors under dietary proline and arginine depletion and/or difluoromethylornithine (DFMO) treatment. a) Heatmap of significantly changed serum metabolites compared to control diet (CD). Metabolites are selected if significantly changed (q < 0.05) in any of the 3 comparisons: Diet effect (ProArg-free vs. CD), DFMO Monotherapy (CD DFMO vs. CD) and Combined Treatment (ProArg-free DFMO vs. CD). Unsupervised clustering performed using Ward’s method. For each intervention, individual mice are displayed relative to the average level in 7 CD mice. *n* = 7-10. b-c) Differential serum metabolite levels in DFMO Monotherapy and Combined Treatment. Blue highlights metabolites that are significantly depleted (FDR < 0.05) and shades of red up regulated in the treatment group compared to CD. *n* = 7-10. d) Tumor levels of arginine, proline, glutamine and ornithine, compared to CD. *n* = 5-6 e) Tumor levels of proline related metabolites dipeptide glycol-l-proline and hydroxyproline, compared to CD. *n* = 5-6. f) Tumor levels of acetylated polyamines, compared to CD. *n* = 4-6. g) Comparison of polyamine levels in tumor tissue upon combining DFMO with diet changes or the polyamine uptake inhibitor AMXT1501. Zooms display differences in polyamine levels between ProArg-free DFMO and AMXT1501 DFMO. Mean ± s.e.m.,Double Diet Amino Acids *n* = 4-5. Uptake inhibition *n* = 8-10. h) Tumor amino acid and ornithine levels in different diets and DFMO co-treatment. ProArg-free combined amino acid depletion enhances ornithine depletion in tumor tissue compared to a single amino acid (Pro-free and Arg-free) diet. Mean ± s.e.m., *n* = 5-12. i) Tumor polyamine levels upon diet change and combination with DFMO show enhanced depletion only in the combined ProArg-free diet and not in the single amino acid depletion arms. Mean ± s.e.m., *n* = 4-5. For a-f: metabolomics was performed on prolonged treatment tumors (average sacrifice at 12 weeks in all arms). d-i: *P < 0.05, **P < 0.01, ***P < 0.001, ****P < 0.0001, two-tailed t-test. Mean ± s.e.m. Technical replicates are averaged for each mouse. Abbreviations: CD, control diet; ProArg-free, proline and arginine-free diet; DFMO, difluoromethylornithine; Pro-free, proline-free diet; Arg-free, arginine-free diet. Source data

To reveal the metabolic reprogramming underlying this anti-tumour activity, we performed serum metabolomics (Fig. 3 and Extended Data Fig. 5a). Across the entire metabolome, the metabolite showing the most significant decrease in serum in response to the ProArg-free diet was ornithine, the crucial polyamine precursor that we sought to deplete. The next two most significantly decreased metabolites were proline and arginine themselves (Fig. 3b,c and Extended Data Fig. 5b,c). Other notable serum metabolite changes included an increase in glutamine and decreases in citrulline and the collagen breakdown product hydroxyproline (Fig. 3b,c and Extended Data Fig. 5c). Arginine, proline and ornithine were also significantly decreased in tumours following different treatment durations, but to a lesser extent than in serum (Fig. 3d and Extended Data Fig. 5d,e). The depletion of intratumoral ornithine manifested despite the increase in tumour glutamine, which gives rise to ornithine in other cancers via OAT^16^.

Targeted liquid chromatography–mass spectrometry (LC–MS/MS) measurements of tumour polyamines revealed that DFMO treatment decreased putrescine, the direct product of ornithine decarboxylation by ODC, and its derivatives such as spermidine. The ProArg-free diet potentiated the DFMO effect to further decrease polyamine levels, achieving more than tenfold reduction in spermidine compared with CD and more than twofold reduction compared with DFMO monotherapy (Fig. 3e). *N*^8^-acetylspermidine and *N*-acetyl-putrescine were also decreased by a ProArg-free diet plus DFMO, consistent with reduced catabolic flux (Fig. 3e and Extended Data Fig. 5f). Combined dietary intervention with DFMO treatment resulted in superior spermidine depletion compared with co-treatment with the polyamine uptake inhibitor AMXT1501^24^ (Fig. 3e and Extended Data Fig. 5g), highlighting the primacy of intratumoral polyamine synthesis. Reducing proline or arginine substrate intake individually was insufficient to enhance polyamine depletion beyond DFMO monotherapy (Extended Data Fig. 5h,i). The dependency on intracellular polyamine biosynthesis of neuroblastoma via ODC and its substrates was further confirmed in an ex vivo neuroblastoma cell model, with depletion of proline and arginine from the medium synergizing when ODC was inhibited (DFMO) or downregulated (via short hairpin RNA (shRNA)) (Supplementary Fig. 2). Growth rescue was observed from natural and synthetic polyamines across neuroblastoma lines upon polyamine biosynthesis inhibition by DFMO or combined with proline and arginine depletion medium (Supplementary Fig. 2h–j). Thus, dual dietary amino acid restriction depletes the key polyamine precursor ornithine and, combined with DFMO, leads to enhanced tumour polyamine depletion.

## Induction of defined translation defects

**Fig. 4:**
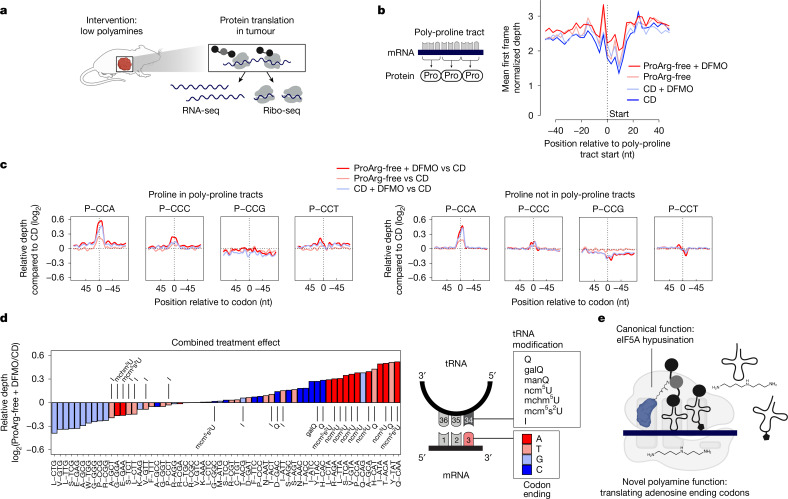
Ribo-seq reveals defective decoding of codons with adenosine in the third position following polyamine depletion. **a**, For functional evaluation of translation, tumours were lysed in the presence of a translation inhibitor for preparation of RNA-seq and Ribo-seq libraries. Ribosome-protected RNA fragments were isolated and sequenced to assess translation. **b**, Normalized ribosome depth at positions encoding three or more consecutive proline residues. Decoding of these polyproline tracts is affected by combining DFMO (1% in the drinking water) with proline and arginine-free diet. **c**, Proline translation defects are codon-specific. Relative ribosome density centred around proline codons across treatment groups relative to CD (zero line). Left, density of ribosomes at polyproline tracts. Right, codon occupancy on proline codons outside of polyproline tracts. Increased occupancy manifests at CCA and less at CCC. **d**, Codons with adenosine in the third position show specific translation defects induced by the combined ProArg-free diet plus DFMO treatment compared with CD diet when comparing the transcriptome-wide relative ribosome occupancy. Codons that require tRNAs with modifications at position 34 for decoding are highly enriched in codon pausing following ProArg-free diet plus DFMO treatment compared with CD. Relative pausing of codons in the ribosomal P site. GalQ, galactosyl-queuosine; I, inosine; manQ, mannosyl-queuosine; mchm^5^U, 5-methoxycarbonyl-hydroxymethyluridine; mcm^5^s^2^U, 5-methoxycarbonylmethyl-2-thiouridine; ncm^5^U, 5-carbamoylmethyluridine; Q, queuosine. **e**, Schematic showing two mechanisms of polyamine depletion therapy. Only the combined treatment induces mild hallmarks of eIF5A hypusination deficiency and boosts the codon-specific translation defect induced by polyamine depletion. As described in Fig. 3, data in **b**–**e** are from the *Th-MYCN* mouse model. For all mean, *n* = 5. Panels **a** and **e** created in BioRender. Morscher, R. (2025) https://BioRender.com/75ofpvn (**a**); https://BioRender.com/iydko99 (**e**). Source data

**Extended Data Fig. 6:**
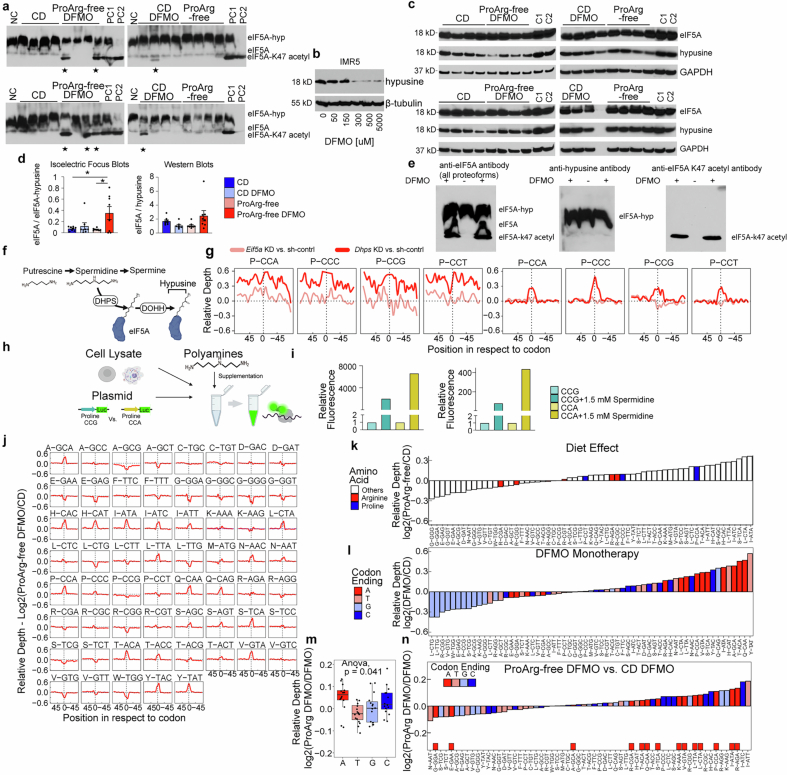
Polyamine depletion causes codon specific translation defects. a) eIF5A hypusination defects detected by isoelectric focusing followed by immunoblotting. Tumors of ProArg-free and CD DFMO arms, showing defective hypusination in only two CD DFMO tumors and five ProArg-free DFMO tumors. Neuroblastoma cell line IMR5 as negative control (PC1), not treated with DFMO, and positive control (PC2), treated for five days with 500 uM of DFMO. Stars denote tumors with a non-hypusinated eIF5A (eIF5A-K47acetyl) band compared to hypusinated band (eIF5A-hyp). b) Neuroblastoma cell line IMR5 (used as positive control in other panels) treated with increasing DFMO concentrations for five days and assessed by immunoblotting with an anti-hypusine antibody. c) eIF5A hypusination defects detected by immunoblotting. Tumors of ProArg-free DFMO show partially reduced hypusination. IMR5 (C1) and CHLA20 (C2) were added as negative controls for comparison to incomplete hypusination. d) Quantification of hypusination by isoelectric blots reported in a) and immunoblots reported in b). Mean ± s.e.m. *P < 0.05, **P < 0.01, ***P < 0.001, ****P < 0.0001, two-tailed t-test. *n* = 7-8 per group. e) Validation of isoelectric blots bands. IMR5 protein lysates treated with 500 uM DFMO for 5 days. In a single IEF gel, these 3 lysates were run in triplicate, the membrane cut, and independently probed without stripping, using anti-eIF5A (detecting all forms), anti-hypusine (detecting only hypusinated eIF5A), and anti-eIF5A-K47 acetyl (detecting only K47-acetylated forms). f) Schematic of eIF5A hypusination via DHPS using spermidine as a substrate. g) Relative ribosome density in relation to proline codons upon *Eif5a* and *Dhps* knock down (KD) as compared to short-hairpin control (sh-contrl). The left panels are centered by poly proline tracts and the right by proline codons outside thereof. In Dhps KD, increased occupancy shows at all proline codons independent of the nucleotide three position (e.g. adenosine-ending). Reprocessed and reanalyzed data from Nakanishi et al. n = 2 per group. h) In vitro translation setup of IMR5 neuroblastoma cell lysates that were harvested in log-phase growth. Lysates were co-incubated with in vitro transcribed mRNA fragments encoding a repeat of either 7 instances of CCA or CCG just 5’ upstream of luciferase. Changes in fluorescent intensity thereby reflect the effect of polyamines on the translation of the codon repeats. i) Polyamine supplementation with 1.5 mM spermidine preferentially facilitates translation of CCA codon repeats, as compared to CCG stretches. Relative fluorescence to baseline translation of two independent experiments are shown. j) Translation defects are codon specific. Relative ribosome density on all amino acid codons in combined drug-diet treatment (ProArg-free DFMO vs. CD). First letter of name denotes amino acid followed by the encoding codon. k) The diet effect (ProArg-free vs. CD) in the ribosomal P site is not driven by pausing at arginine and proline amino acid codons. l) DFMO treatment effect (CD DFMO vs. CD) is characterized by ribosome pausing depending on the nucleotide at the codon-three position in ribosome P site. m) Adding a ProArg-free diet to DFMO treatment is characterized by enhanced ribosome pausing depending on the nucleotide identity at the codon-three position in ribosomal P site (ProArg-free DFMO vs. CD DFMO). Boxplot where the center line represents the median, the box spans the interquartile range (IQR; 25th to 75th percentiles), and whiskers extend to 1.5I QR. Data points beyond this range are shown as outliers (solid black). One-way ANOVA. A-ending codons *n* = 14, T-ending codons *n* = 16, G-ending codons *n* = 15, C-ending codons *n* = 16. n) Increased pausing at A-ending codons when comparing ProArg-free DFMO vs. CD DFMO at the ribosomal P site. j-n shows mean of *n* = 5. Abbreviations: CD, control diet; ProArg-free, proline and arginine-free diet; DFMO, difluoromethylornithine. Panels f and h created in BioRender. Morscher, R. (2025) https://BioRender.com/sf37unt (f); https://BioRender.com/7ngicj2 (h). Source data

Polyamines stimulate translation and cell growth^26^. Arginine and proline also feed directly into translation as proteinogenic amino acids. Combining proline and arginine depletion with DFMO in vitro enhanced translation inhibition by DFMO (Supplementary Fig. 3a–c). To disentangle the amino acid and polyamine effects of the ProArg-free diet and DFMO on translation, we carried out ribosome profiling (Ribo-seq)^27^ (Fig. 4a; quality control presented in Supplementary Fig. 3d–h). This approach identifies transcriptome-wide mRNA loading with ribosomes and which codons are decoded by ribosomes at the moment of cell lysis. Increased ribosome density can indicate sites of stalled translation, due either to uncharged tRNAs or a defect in the translation machinery. With ProArg-free diet, DFMO or ProArg-free diet plus DFMO, ribosome occupancy was shifted slightly towards the start codon and decreased at early elongation of the protein-encoding transcript. Exclusively under ProArg-free diet plus DFMO treatment, ribosomes accumulated at stop codons, indicating defective ribosome release (Supplementary Fig. 3i,j). Such elongation and termination defects have been reported in cell models functionally deficient in eIF5A^28^, a translation factor that is post-translationally modified by the polyamine spermidine^27,29–31^. As this suggested in vivo eIF5A dysfunction, we probed eIF5A hypusination status (that is, spermidine modification of eIF5A) across treatment groups. Whereas all tumours from the CD and ProArg-free groups without drug demonstrated complete eIF5A hypusination, reflecting sufficient spermidine for this purpose, two out of eight tumours treated with DFMO alone and five out of eight tumours from the ProArg-free diet plus DFMO group had reduced eIF5A hypusination and increased K47 eIF5A acetylation, indicative of attenuated hypusination. We also observed modestly reduced hypusination in these tumours using hypusine-specific antibodies (Extended Data Fig. 6a–e).

In addition to its global functions, hypusinated eIF5A has been implicated in facilitating peptide bond formation involving repetitive instances of the amino acid proline, termed polyproline tracts. Owing to its reactive amine localized within a ring structure, proline is a poor peptidyl acceptor^32^. We thus evaluated relative ribosome occupancy at polyproline tracts, with high occupancy indicating slow decoding by ribosomes, due either to uncharged proline tRNA or to eIF5A deficiency. Increased occupancy was observed at polyproline tracts in ProArg-free diet plus DFMO-treated tumours, but not at proline codons with ProArg-free diet alone (Fig. 4b). Collectively, these data indicate that the combined ProArg-free diet and DFMO therapy impairs translation beyond tumour amino acid levels and tRNA charging by depleting spermidine to levels low enough to also impair eIF5A hypusination in more than half of the tumours.

## Slow decoding of codons ending with adenosine

As the role of polyamine levels in ribosomal decoding at codon resolution is currently unknown, we next sought to differentiate its effects from eIF5A hypusination-related roles in translational reprogramming. Further analysis of the Ribo-seq data revealed a surprising aspect of the stalling at polyproline tracts induced by combined ProArg-free diet and DFMO therapy: ribosome stalling was observed predominantly at only one of the four proline codons (CCA). There was less stalling at CCC and no stalling at CCG or CCT. This indicated an additional unanticipated level of translation regulation at the codon, rather than the amino acid level. Extending the analysis to all proline codons (that is, including those that were not in polyproline tracts) revealed the same phenomenon: selected stalling at CCA codons (Fig. 4c). This phenotype contrasts with the genetic modulation of eIF5A hypusination, which preserves normal polyamine levels, yet affects all proline codons (Extended Data Fig. 6f,g). Conversely, supplementing neuroblastoma cell lysates with polyamines in an in vitro translation assay preferentially facilitated the translation of CCA codons over CCG codons (Extended Data Fig. 6h,i). Together, these results highlight an effect of polyamine depletion that is not amino acid-specific or recapitulated by genetic ablation of hypusination as the dominant driver of codon-specific translational reprogramming under ProArg-free diet plus DFMO treatment.

We next assessed the global effect of combined ProArg-free diet plus DFMO treatment across individual codons at high resolution. Across all amino acids, ribosome pausing was highly dependent on the codon type (as opposed to amino acid identity). When sorting individual codons by relative occupancy, a primary factor determining translation speed was the nucleotide at the third position. Whereas codons with adenosine in the third position showed increased occupancy (a proxy for stalling) in ProArg-free diet plus DFMO-treated tumours, occupancy at codons with guanosine in the third position was markedly decreased (Fig. 4d and Extended Data Fig. 6j). The same phenotype, with a reduced effect size, was shown for the DFMO monotherapy group, suggesting a previously unknown role of polyamines (rather than low arginine or proline) in the decoding of codons with adenosine at the third position (also referred to as the ‘wobble’ position) (Fig. 4e and Extended Data Fig. 6k–n). Similarly, at the global level this phenotype was not identified following genetic or pharmacological inhibition of hypusination (Supplementary Fig. 4).

On the contrary, ribosome pausing appeared to be driven by disrupted codon–anticodon interactions with tRNAs exhibiting complex biochemical modifications at the anticodon wobble base position (tRNA base 34) of all highly paused codons with adenosine in the third position, as well as the two highly paused codons with thymidine in the third position (Fig. 4d). This was further supported by the pausing phenotype affecting all three ribosome sites (A, P and E; Extended Data Fig. 6j). Whereas this was mostly independent of the tRNA modification status at position 34, the ProArg-free diet itself increased queuosine-related modifications, putatively further adding to the polyamine induced codon-specific translation imbalance (Supplementary Fig. 5a–d). Although interactions of polyamines with ribosomes and tRNAs have been reported^33,34^ and documented to stimulate translation in vitro^35^, to our knowledge, this codon resolution phenotype has not previously been described (Fig. 4e).

## A codon-specific proteome rewiring

**Fig. 5:**
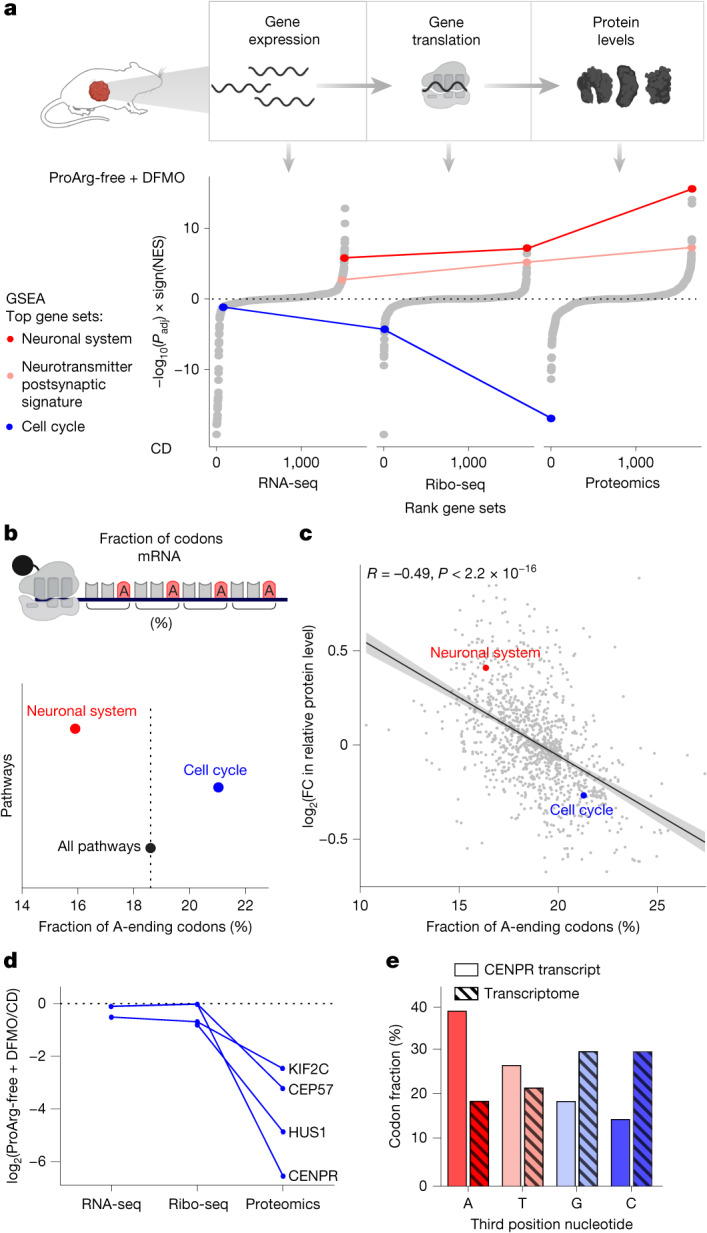
Regulation of translation by polyamine depletion is driven by fractional codon content. **a**, Gene set enrichment analysis (GSEA) of protein biosynthesis using omics layers: gene-expression (RNA-seq), gene translation (Ribo-seq) and protein (proteomics) levels. GSEA compares ProArg-free plus DFMO to CD using the Reactome gene sets. All pathways are depicted, ranked by significance (Benjamini–Hochberg correction) and signed by normalized enrichment score (NES). The most downregulated and upregulated sets at the protein level are cell cycle and neuronal system, respectively. Lines connect gene sets across the omics layers. **b**, Mean fraction of codons with adenosine in the third position (A-ending codons) across all pathways and significantly changed pathways identified in **a**. Pathways taken from Reactome gene sets. **c**, The percentage of codons with adenosine in the third position correlates with the average protein level across Reactome pathways. Pathways with an increasing fraction of codons with adenosine in the third position have lower protein levels in ProArg-free DFMO compared with CD. FC, fold change. **d**, Fold change across omics layers of top downregulated cell cycle proteins indicates that differences between ProArg-free diet plus DFMO and CD occur predominantly on the protein level. **e**, Percentage of codons with the respective nucleotide at the third position in the *Itgb3bp* gene (encoding CENPR protein) compared with the transcriptome background. In **a**,**c**,**d**, RNA-seq, ProArg-free + DFMO: *n* = 5; CD: *n* = 4. Ribo-seq, *n* = 5. Proteomics, ProArg-free + DFMO: *n* = 6; CD: *n* = 5. Panels **a** and **b** created in BioRender. Morscher, R. (2025) https://BioRender.com/ygrgncb (**a**); https://BioRender.com/566gynw (**b**). Source data

To better understand the phenotypic consequences induced by this specific translation defect, we integrated RNA-sequencing (RNA-seq), Ribo-seq and proteomics data (Fig. 5a and Supplementary Fig. 6a–d). The main driver of transcriptional changes was DFMO. The ProArg-free diet itself induced only minor such changes, and transcriptional changes strongly overlapped between DFMO alone and the combined ProArg-free diet plus DFMO treatment. By contrast, Ribo-seq and proteomics revealed distinct changes in tumours under combined ProArg-free diet plus DFMO therapy. The effect was independent of proline or polyproline abundance in the proteins (Supplementary Fig. 6e–h), and instead correlated with genes that were enriched in codons with adenosine in the third position.

**Extended Data Fig. 7:**
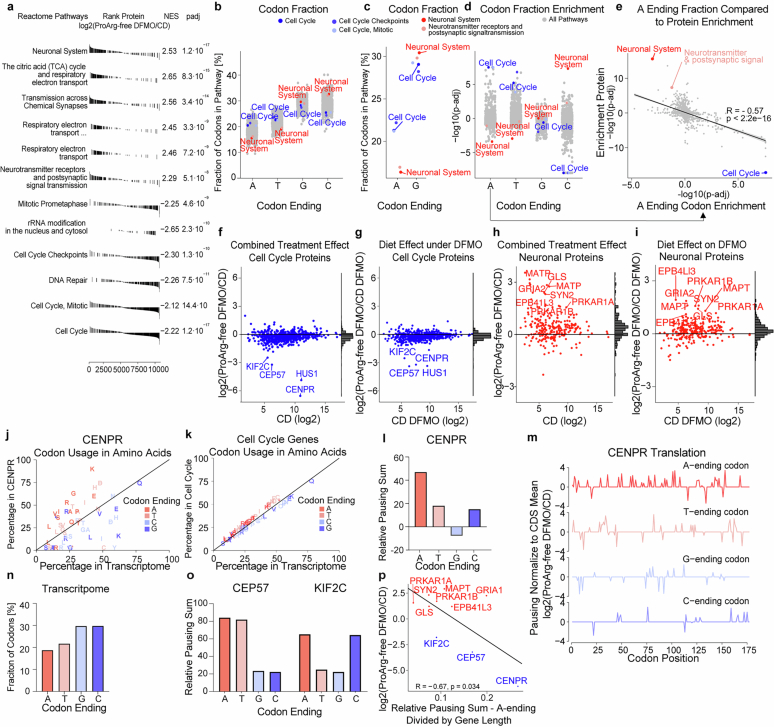
Adenosine-ending codon frequency correlates with translation defects. a) Top down- and upregulated Reactome pathways in the GSEA analysis on the protein level when comparing the diet-drug combination to the control diet (ProArg-free DFMO vs. CD). b) Fraction of codons ending with respective nucleotide across all Reactome pathways (grey), cell cycle related (blue) and neuronal system (red). c) The fraction of A-ending codons is more divergent than the fraction of G-ending codons in cell cycle related (blue) and neuronal system (red) emphasizing A-ending codon content as a differential metric for regulating translation of these two pathways. d) Enrichment analysis based on the A, T, G or C ending codon fraction per gene. Pathways positively enriched (positive p-adj) have higher A, T, G or C ending fractional enrichment whereas negatively enriched pathways have a lower fraction. e) Across Reactome pathways, increasing enrichment of adenosine-ending codons correlates to downregulation on the protein level when comparing combined diet-drug treatment to control. Enrichment was computed using gene set enrichment analysis, where transcripts are ranked by adenosine-ending codon fraction and proteins by fold change on the protein levels. f) Protein intensity across cell cycle, the most downregulated pathway, where the difference between ProArg-free DFMO and CD is evaluated by protein fold-change on the y axis. Fold change distribution on the right side. g) ProArg-free DFMO and CD DFMO highlighting the additive diet effect on the downregulation of cell cycle proteins. Four top-down regulated proteins were identified in both comparisons. Fold change distribution on the right side. h) Protein intensities across the most upregulated pathway, neuronal system, where the difference between ProArg-free DFMO and CD is evaluated by protein fold-change on the y axis. Fold change distribution on the right side. i) ProArg-free DFMO and CD DFMO highlighting the additive diet effect on the upregulation of neuronal system proteins. Common top-upregulated proteins (log2 fold change >1) were identified in both comparisons. Fold change distribution on the right side. j) Relative codon preference comparing the *Itgb3bp* gene (CENPR protein) to the whole transcriptome. Percentages above the line highlight a preferential use for encoding the amino acid in *CENPR* by that specific codon type (red is adenosine ending). k) Relative amino acid codon preference comparing the pathway cell cycle to the whole transcriptome. Marks above the line highlight the preferential utilization of defined codons in the cell cycle genes to encode the same amino acid. l) The relative ribosome pausing sum as the sum of ribosome occupancy ratios of ProArg-free DFMO to CD according to the nucleotide at codon position three in *Itgb3bp* (CENPR protein). Specific dysfunction in decoding of A-ending codons is observed. m) Relative ribosome occupancy across *Itgb3bp* (CENPR protein) reveals a dysfunction in decoding adenosine-ending codons. Occupancy ratios were calculated between ProArg-free DFMO and CD. Summed by the nucleotide at codon position three in i). n) Percentage of codons with the respective nucleotide at the ending position in the whole transcriptome. o) Relative pausing sum of *Cep57* and *Kif2c*, where the relative ribosome occupancy ratio between ProArg-free DFMO and CD are summed according to the nucleotide at the codon ending position. *Hus1* did not show sufficient coverage. p) Correlation of relative pausing sum with protein levels in the top up and down regulated proteins. The relative ribosome occupancy ratio between ProArg-free DFMO and CD are summed according to the nucleotide at the codon ending position and normalized for gene length. For a, d, f, g, h, i and p: Proteomics ProArg-free DFMO *n* = 6; CD DFMO *n* = 6; CD *n* = 5. For l, m, o, and p: Ribo-Seq *n* = 5 per group; For b-e, the legend for pathways is shared above. Abbreviation: ‘Respiratory electron transport …’, ‘Respiratory electron transport; ATP synthesis by chemiosmotic coupling, and heat production by uncoupling proteins ‘; NES, Normalized Enrichment Score. Source data

Notably, upon combined ProArg-free diet plus DFMO treatment, gene sets linked to neuronal differentiation and neuronal cell identity were the most upregulated at the protein level. By contrast, ‘cell cycle’ was the most downregulated gene set, despite exhibiting unchanged RNA expression (Fig. 5a and Extended Data Fig. 7a). We next assessed whether the slow translation of codons with adenosine at the third position relates to proteome reprogramming. Indeed the cell cycle gene set contained a higher frequency of codons with adenosine in the third position that were affected by ribosome pausing, compared with the average for the exome. Exome-wide, pathways associated with cell cycle programmes were the most enriched in codons with adenosine in the third position^36^. Neuronal differentiation-associated gene sets were at the opposite end of the spectrum, with the guanosine content showing limited variation in both gene sets (Fig. 5b and Extended Data Fig. 7b–d). A higher fraction of codons with adenosine in the third position within a pathway was linked with a lower protein fold change or gene set enrichment (Fig. 5c and Extended Data Fig. 7e).

**Extended Data Fig. 8:**
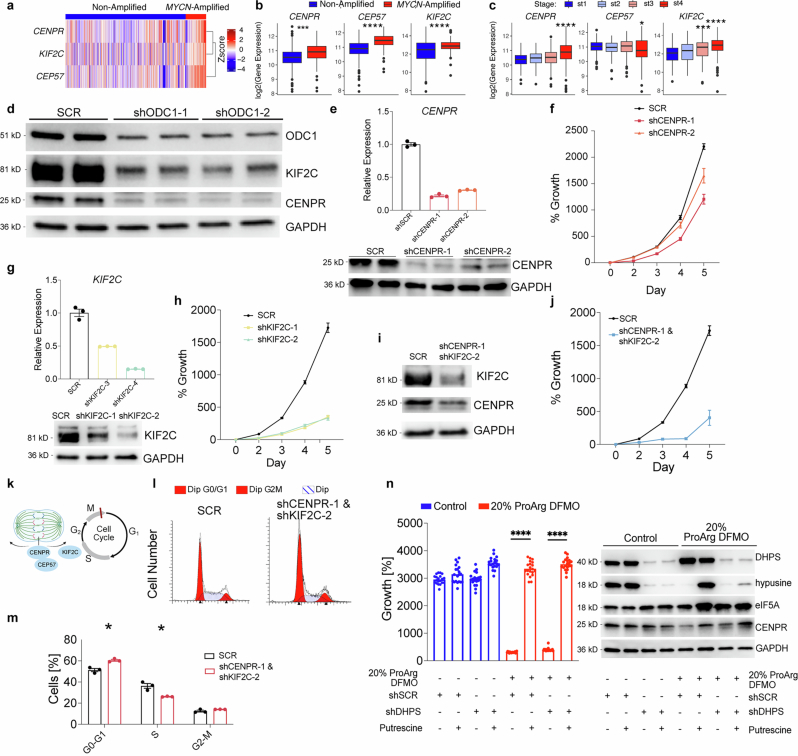
Downregulated cell cycles proteins identified in vivo contribute to growth reduction in vitro*.* a) Heatmap of gene expression from cell cycle genes of interest according to MYCN status highlights their overexpression in *MYCN* amplified primary tumors (*n* = 93), as compared to their non-amplified counterpart (*n* = 551). b) The three cell cycle genes are overexpressed in relation to MYCN status (non-amplified *n* = 551, amplified *n* = 93). c) CeNPR, KIF2C and CEP57 show a stage dependent expression profile with an increased expression in high-risk neuroblastoma. Stage 4 special (st4s) was removed for simplicity. Statistical comparison to stage 1. Number of patients per stage: st1 = 152, st2 = 113, st3 = 90, st4 = 211. d) Cell cycle proteins are concomitantly reduced upon ODC1 knock down (KD) using two short hairpin (sh) compared to scramble (SCR) in IMR5 neuroblastoma cell line. e) Relative expression of CENPR upon KD with two independent sh-CENPR to SCR in IMR5 and the confirmation of reduced protein levels on western blot. Mean ± s.e.m. *n* = 3 per group. f) Reduction of growth upon CENPR KD with two sh-CENPR constructs as compared to SCR. *n* = 16 measurements per day per condition. g) Relative expression of KIF2C upon KD with two independent sh-KIF2C as compared to SCR. And the confirmation of reduced protein levels on western blot. Mean ± s.e.m. *n* = 3 per group. h) Reduction of cell growth upon KIF2C KD compared SCR in the IMR5 neuroblastoma cell line. *n* = 10 measurements per day per condition. i) Western blot confirming reduced KIF2C and CENPR protein levels upon combined KD with sh-CENPR and sh-KIF2C. j) Cell growth upon combination of CENPR and KIF2C KD. *n* = 10 measurements per day per condition. k) Schematic of the involvement of the respective cell cycle proteins of interest S to in G2 phase transition as components of the CENPA-CAD complex. After being recruited to centromeres, they are involved in assembly of kinetochore proteins, mitotic progression and chromosome segregation. l) Cell cycle phase distribution as evaluated using flow cytometry of scrambled and combined sh-CENPR and sh-KIF2C KD. m) Concomitantly to a G0-G1 increase upon combined knock down of CENPR and KIF2C S-phase is decreased. Statistical comparison to SCR. Mean ± s.e.m. *n* = 3 per group. n) Decoupling hypusination dependent and independent polyamine effects. Inhibition of polyamine dependent hypusination via genetic knock down of *Dhps* combined with intracellular polyamine depletion by 20%ProArg media with 500uM DFMO. In neuroblastoma putrescine supplementation (100uM) rescues growth rates and protein levels as demonstrated by immunoblotting without rescuing hypusination of eIF5a. Mean ± s.e.m. *n* = 19-20 per group. Abbreviations: sh, short hairpin; ODC1, ornithine decarboxylase 1; KD, knock down; SCR, scramble; Pro, proline; Arg, arginine; DFMO, difluoromethylornithine. For b and c: Boxplot where the center line represents the median, the box spans the interquartile range (IQR; 25th to 75th percentiles), and whiskers extend to 1.5 IQR. Data points beyond this range are shown as outliers (solid black). For b, c, m and n: two-tailed t-test; *P < 0.05, **P < 0.01, ***P < 0.001, ****P < 0.0001. Panel k created in BioRender. Morscher, R. (2025) https://BioRender.com/u25yr73. Source data

Beyond the general reduction of cell cycle proteins, four mitosis-related proteins stood out with the strongest downregulation at the protein level under combined ProArg-free diet plus DFMO treatment (Extended Data Fig. 7f,g). All four showed at least fourfold downregulation at the protein level, without changes in gene expression (Fig. 5d), in contrast to the regulation in neuronal proteins (Extended Data Fig. 7h,i). *Itgb3bp*, encoding CENPR, the most affected protein, showed a remarkably shifted distribution in codon preference, with 38.6% of codons having adenosine in the third position, compared with 18.8% across all protein-coding transcripts (Fig. 5e and Extended Data Fig. 7j,k). The ribosome distribution along the CENPR coding sequence confirmed preferential pausing at codons with adenosine in the third position upon ProArg-free diet plus DFMO treatment, as indicated by an increased ribosomal pausing sum at codons with adenosine in the third position and reduced pausing at those with guanosine in the third position (Extended Data Fig. 7l–n). Similar pausing was observed for CEP57 and KIF2C, and the pausing sum correlated to the levels of the most regulated proteins across the two gene sets (Extended Data Fig. 7o,p). Expression of cell cycle proteins was increased in *MYCN*-amplified tumours from human patients and correlated to high tumour stage (Extended Data Fig. 8a–c). Depleting polyamine levels independently of DFMO by ODC knockdown depleted cell cycle protein levels and induced growth defects characterized by cell cycle arrest (Extended Data Fig. 8d–m). Conversely, external supplementation of polyamines restored cell growth and CENPR levels following enhanced polyamine depletion by DFMO with proline- and arginine-reduced medium. This rescue was independent of hypusination status, as indicated by sustained rescue upon *Dhps* knockdown, preventing spermidine-dependent post-translational modification of eIF5a (Extended Data Fig. 8n). Thus, polyamine depletion by combined ProArg-free diet and DFMO treatment induces ribosome stalling at codons with adenosine in the third position. Owing to the selective enrichment of these codons cell cycle genes and their depletion in differentiation genes, this effect of ProArg-free diet and DFMO treatment reprogrammes the proteome in a manner that favours differentiation.

## Therapeutic differentiation of neuroblastoma

**Fig. 6:**
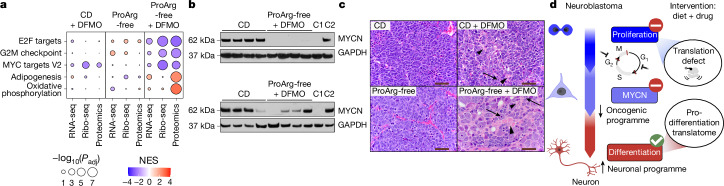
Polyamine depletion-mediated proteome rewiring induces neuroblastoma differentiation. **a**, GSEA across omics layers in all three treatment groups using the Hallmark gene set. Only the ProArg-free plus DFMO treatment group showed a significant effect compared with CD. The effect was mainly on the translation and protein level. Shown are the five top enriched sets (complete sets in Extended Data Fig. 10c). Size indicates *P* value (Benjamini–Hochberg correction) and colour represents NES, with red indicating enrichment in the intervention group (CD + DFMO, ProArg-free or ProArg-free + DFMO) and blue indicating enrichment in the CD group. **b**, Western blot analysis of MYCN in tumours from CD and ProArg-free plus DFMO treatment arms. Negative control (C1), CHLA20 neuroblastoma cell line (*MYCN* non-amplified, MYC expressing); positive control (C2), IMR5 neuroblastoma cell line (*MYCN-*amplified). GAPDH is used as a loading control. Blots are representative of two independent experiments yielding similar results. **c**, Representative haematoxylin and eosin (H&E)-stained sections. CD and ProArg-free diet treatments show undifferentiated primitive neuroblasts, absent neuropil and prominent mitotic figures. CD plus DFMO shows poorly differentiated primitive neuroblasts with scant neuropil (arrowhead) and foci of cytodifferentiation (<5% differentiating, arrow). ProArg-free diet plus DFMO tumours show high fractions of differentiating neuroblasts (>5% differentiating) with increased cytoplasmic to nuclear ratio (arrow) and abundant neuropil (arrowhead). Sections are representative of many images with  the same observations. Scale bars, 50 μm. **d**, Summary of treatment effects. Cell cycle and MYCN programmes are downregulated at the protein level owing to translation inhibition and immature cancer cells are driven into neuronal differentiation. In **a**,**c**, RNA-seq: ProArg-free DFMO: *n* = 5; CD: *n* = 4. Ribo-seq: *n* = 5. Proteomics: ProArg-free DFMO: *n* = 6; CD: *n* = 5. Panel **d** created in BioRender. Morscher, R. (2025) https://BioRender.com/kk9n051. Source data

**Extended Data Fig. 9:**
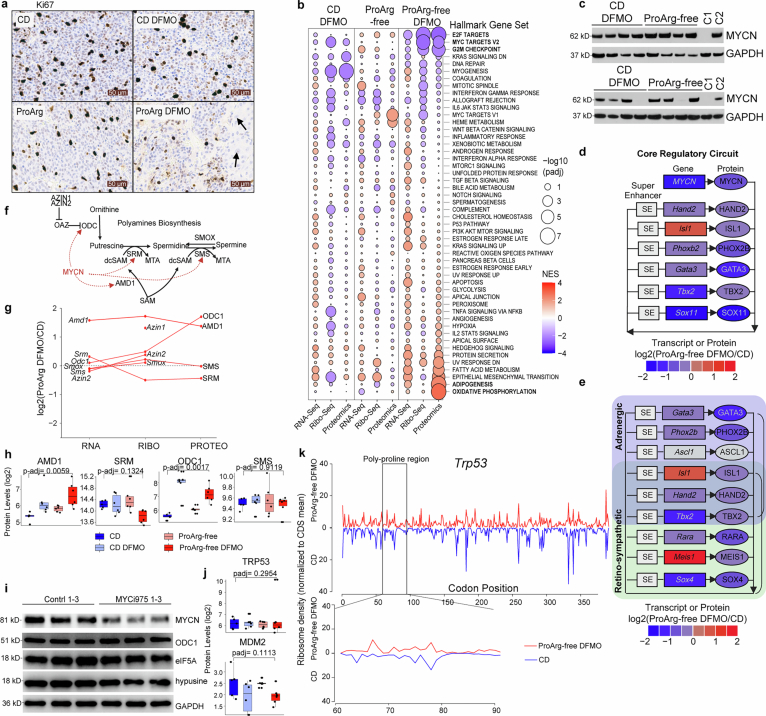
Targeting metabolic dependencies of translation affects hallmarks of cellular regulation. a) Immunohistochemistry showing Ki67 (proliferation marker) of representative *Th*-*MYCN* tumors. Arrows denote cells in areas with local cytodifferentiation. b) Hallmark gene set enrichment across omics layers. Displayed is the full gene set tested across each treatment group CD DFMO, ProArg-free and combined ProArg-free DFMO compared to CD. The top 5 significantly changed gene sets on protein level are highlighted in bold. Point size denotes the significance level and the color scale the normalized enrichment score (NES), with red showing enrichment in the intervention group (CD DFMO, ProArg-free or ProArg-free DFMO) and blue in CD. c) Western blot analysis of MYCN in tumors from CD DFMO and ProArg-free treatment arms. Control 1(C1) is CHLA20, NB cell line with MYC amplification (no MYCN expressed) C2 is control IMR5, NB cell line with MYCN amplification and high expression. d) Combined diet-drug treatment disrupts the *MYCN*-driven super enhancer core regulatory circuitry on the transcript expression (square), and the protein level (ellipsoid). Similarly, other elements of the core regulatory circuitry are affected. e) Combined diet-drug treatment disrupts the *MYCN-*driven super enhancer adrenergic and retino-sympathetic regulatory circuitry on the transcript expression (square), and the protein level (ellipsoid). f) The polyamine biosynthetic pathway with targets reported to be regulated by MYCN on the transcriptional level highlighted by red arrows. g) Regulation of core polyamine biosynthesis pathway members given by fold change across the omics layers. Despite MYCN loss on the protein level, core targets including AMD1 (RNA level) and ODC1 (protein level) are upregulated. Others are broadly unchanged. h) Levels of enzymes in polyamine biosynthesis across treatment groups. Adjusted p-value is from the comparison between CD and ProArg-free DFMO. i) Western blot analysis of neuroblastoma cell line IMR5 treated with 5 uM MYCi975 for four days show downregulation of MYCN, no change of ODC1 and eIF5A hypusination. j) The mouse orthologue for TP53, TRP53 and its core regulator MDM2 are unchanged across all four treatment groups in the *Th*-*MYCN* neuroblastoma tumors (CD, CD DFMO, ProArg-free or ProArg-free DFMO). k) Ribosome density along the TRP53 coding sequence normalized to CDS mean. Insert highlights the proline rich region with no signs of ribosome stalling at the respective proline codons in the ProArg-free DFMO combination treatment. For c, d and e RNA-Seq: CD DFMO, ProArg-free and ProArg-free DFMO n = 5; CD n = 4, Ribo-Seq: n = 5 per group, Proteomics: CD DFMO, ProArg-free and ProArg-free DFMO n = 6; CD n = 5. For h and j: Boxplot where the center line represents the median, the box spans the interquartile range (IQR; 25th to 75th percentiles), and whiskers extend to 1.5 IQR. Data points beyond this range are shown as outliers (solid black). CD DFMO, ProArg-free and ProArg-free DFMO *n* = 6; CD *n* = 5. Abbreviations: CD, control diet; ProArg-free, proline arginine free diet; DFMO, difluoromethylornithine; c-d: Reprocessed and reanalyzed data from Elkon et al. *n* = 2 per group. e and f: Reprocessed and reanalyzed data from Volegova et al. *n* = 2 per group. b,i and j: Data generated as part of this study. *n* = 3 per group. Source data

We hypothesized that the shift away from cell cycle proteins towards neuronal differentiation proteins is likely to reprogramme neuroblastoma tumours in a manner that slows proliferation and induces differentiation. Ki67 staining confirmed a decrease in actively cycling cells under ProArg-free diet plus DFMO treatment (Extended Data Fig. 9a). Furthermore, cell growth activation signatures including MYC and E2F targets were impaired (Fig. 6a and Extended Data Fig. 9b).

**Extended Data Fig. 10:**
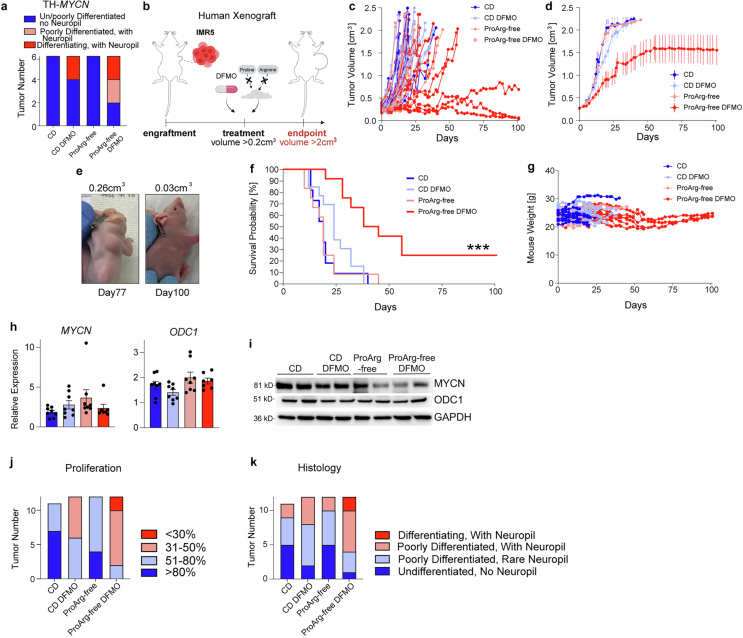
Proline and arginine amino acid depletion effect with DFMO in human xenografts in vivo. a) Blinded histological assessment by a pathologist of differentiation status and abundance of neuropil status in tumor sections (linked to Fig. 6c) in *Th*-*MYCN* mice. *n* = 6 per group. b) Schematic of xenografts induced in nude mice by the MYCN-amplified human neuroblastoma cell line IMR5. Upon reaching 200 mm^3^ mice were randomized to receive either a CD or ProArg-free diet with or without DFMO drug application via the drinking water (1 %). c) Tumor volume of individual xenografts in mice across treatment groups. d) Average neuroblastoma xenograft tumor volume according to the four treatment groups CD, CD DFMO, ProArg-free and ProArg-free DFMO. Upon reaching the maximal tumor volume in an individual tumor (2 cm^3^) this measurement was counted towards the mean until death of the last mouse in this group. Mean ± s.e.m. e) Example of neuroblastoma tumor regression in a ProArg-free DFMO treated mouse (day 77 compared to day 100). f) Kaplan-Meier survival curve related to b) with death encoded for human endpoints or tumor volume above 2 cm^3^. g) Mouse weight corrected for tumor weight across time after engraftment of IMR5 xenografts. h) qPCR quantification of *MYCN* and *ODC1* gene expression in IMR5 xenograft tumors across treatment groups. n = 7-8 per group. Mean ± s.e.m. i) Western blot analysis of MYCN and the DFMO target ODC1 and in IMR5 xenograft tumors after treatment. j) Proliferation rate as assessed by immune histochemistry staining (Ki67 staining) on tumor sections of the four treatment groups. Data acquired by a pathologist blinded to treatment groups. k) Differentiation status according to clinical-diagnostic standards and scoring for neuropil abundance in H&E-stained histology sections derived from the IMR5 intervention trial. Data acquired by a pathologist blinded to treatment groups. Abbreviations: CD, control diet; ProArg-free, proline arginine free diet; DFMO, difluoromethylornithine. For b-h IMR5 xenograft model: CD *n* = 11, CD DFMO *n* = 13, ProArg-free *n* = 12, ProArg-free DFMO *n* = 12. For f: Log-rank test p-value compared to CD. *P < 0.05, **P < 0.01, ***P < 0.001, ****P < 0.0001. For h: two-tailed t-test compared to CD. *P < 0.05, **P < 0.01, ***P < 0.001, ****P < 0.0001. Panel b created in BioRender. Morscher, R. (2025) https://BioRender.com/sd1cd2k. Source data

Given the essential role *of MYCN* in the development and maintenance of neuroblastoma and its positive feedback loop with ODC1 and eIF5A^37^, we explored whether the loss of MYC targets indicates a disruption of the core oncogenic regulatory circuit^38–40^. Both *MYCN* mRNA expression and MYCN protein were preferentially downregulated in tumours under combined ProArg-free diet plus DFMO treatment (Fig. 6b and Extended Data Fig. 9c). Other core transcription factors were also suppressed, supporting a broad disruption of the MYCN-driven core regulatory circuit (Extended Data Fig. 9d,e). Despite MYCN downregulation, its transcriptional targets in the polyamine pathway remained unchanged or were upregulated in response to polyamine depletion in vivo (Extended Data Fig. 9f–h) or pharmacological inhibition in vitro (Extended Data Fig. 9i). Further, *TP53* expression^41^ remained unaffected on the protein or ribosome level (Extended Data Fig. 9j,k). On the mechanistic level, modulating MYC or MYCN activity by genetic or pharmacological means showed no pausing of ribosomes at codons with adenosine in the third position, suggesting a MYC-independent translation phenotype as a driver (Supplementary Fig. 7a–h). Evaluating tumour differentiation status according to clinical pathological criteria showed that tumours in the CD and ProArg-free groups were uniformly undifferentiated (<5% cytologic differentiated with absent neuropil). By contrast, we observed a strong differentiation phenotype upon polyamine-depleting treatment, with one-third of CD plus DFMO-treated tumours being differentiated (more than 5% cytologic differentiated with absent neuropil) and two thirds of ProArg-free diet plus DFMO-treated tumours differentiating or partially differentiating with abundant neuropil (a feature of neural differentiation; Fig. 6c and Extended Data Fig. 10a). Thus, combining a ProArg-free diet with DFMO therapy led to marked reductions in polyamines, inducing selective translation defects that suppressed tumour cell proliferation and induced tumour differentiation (Fig. 6d).

## ProArg-free DFMO in human neuroblastoma

The therapeutic relevance of combining dietary amino acid depletion with DFMO was further emphasized in a model using established patient-derived neuroblastoma cell line xenografts in mice (Extended Data Fig. 10b–e). Replicating the genetic model, long-term survival beyond 100 days and apparent cures were observed in one-quarter of the ProArg-free diet plus DFMO-treated mice and treatment was well tolerated without weight changes (Extended Data Fig. 10f,g). Whereas on the gene-expression level *MYCN* and *ODC1* were unchanged, MYCN protein expression was decreased independently of ODC1 (Extended Data Fig. 10h,i). Mechanistically decreased proliferation was confirmed by histology, along with a pro-differentiation phenotype (Extended Data Fig. 10j,k), highlighting the role of enhanced polyamine depletion inducing differentiation for treatment of neuroblastoma.

## Discussion

Here we find that neuroblastoma, a highly malignant childhood cancer, is vulnerable to polyamine depletion achieved by the combination of a diet free of proline and arginine to deplete the polyamine precursor ornithine, and pharmacological inhibition of ODC, the committed step of polyamine synthesis, with high-dose DFMO. The diet plus drug combination markedly enhanced polyamine depletion and exerted a strong anti-cancer effect in a highly lethal transgenic mouse neuroblastoma model, and a human neuroblastoma mouse xenograft model, with durable complete responses in both.

Use of defined diet and drug combinations is emerging as a clinically viable strategy for cancer treatment^42,43^. Ketogenic diet can synergize with classical chemotherapy and targeted agents, and can be achieved, with proper support, by patients making careful food choices^44–46^ (NCT05300048 and NCT01535911). Diets lacking certain amino acids require laboratory formulation, but show acceptable taste and desired metabolic effects in humans, and are also entering cancer efficacy trials^47,48^ (NCT05078775). Such diets show effects resembling enzyme-based treatments that catabolize particular amino acids, such as asparaginase, a long-standing standard of care for treating childhood leukaemias^49^. In some cases, however, diets have important advantages. For example, dietary arginine depletion decreases both arginine and ornithine, whereas arginase therapy^50^ depletes arginine by converting it into the polyamine precursor ornithine. Conversely, refeeding has been shown to induce polyamine biosynthesis in intestinal stem cells, triggering tumorigenicity^51^. Thus, preventing intestinal interconversion of substrates through a dietary approach or alternative interventions suitable for achieving ornithine depletion offer an effective combination with DFMO to deplete polyamines.

Arrested cellular differentiation by retained embryonal gene-expression circuits is a hallmark of paediatric cancers^52,53^. Neuroblastoma is a prime example, with hyperactive MYCN driving embryonal programmes^38,40,54,55^. Induction of differentiation through transcriptional reprogramming is a validated therapeutic strategy, exemplified by the nuclear hormone receptor agonist retinoic acid^56,57^. Here we provide evidence for the feasibility of triggering differentiation in paediatric cancers through proteome reprogramming. Specifically, we find that polyamine depletion promotes the translation of pro-differentiation proteins and suppresses that of cell cycle proteins, leading to neuronal differentiation of neuroblastoma. Whether similar benefits could be achieved in other MYC-driven cancers merits investigation.

The selective effect of polyamine depletion on translation of certain genes was unexpected, and was not driven by genetic ablation of eIF5A hypusination. The underlying biochemistry involves polyamine deficiency shifting codon optimality by impairing translation of codons with adenosine at the wobble base by position 34-modified tRNAs, and codons being thus preferentially enriched (or depleted) in different gene sets. This mechanism raises the possibility that codon usage has evolved in tandem with metabolism, such that metabolic limitation acts on translation of specific codons to rewire the proteome. Specifically, our data point to polyamine depletion suppressing proliferation and promoting differentiation via utilization of codons with adenosine in the third position. This programme may have evolved to support proper developmental decisions, but using diet and pharmacology, it has therapeutic potential in treating neuroblastoma.

## Methods

### Primary neuroblastoma patient samples

Flash-frozen primary neuroblastoma tumour samples were provided by the Children’s Oncology Group (COG) under study number ANBL16B2 Q. International Neuroblastoma Pathology Classification histologic parameters, *MYCN* amplification status, age and stage for every patient was obtained centrally via the COG Statistics and Data Center. Tumour cell content of samples was confirmed over 80% percent. Patient and tumour characteristics are given in Supplementary Table 1. Water-soluble metabolites were extracted and analysed as described below.

### Mouse models

Animal studies followed protocols approved by Princeton University and Children’s Hospital of Philadelphia Institutional Animal Care and Use Committees. For xenografts used for metabolomics, cancer cell lines were grown in RPMI supplemented with 10% FBS and 0.01% insulin/transferrin solution. Cell lines were provided by the COG Cell Culture Repository: LA-N-5, SMS-SAN, CHLA-90 and SK-N-SH. All cell lines repeatedly tested negative for Mycoplasma. Subcutaneous xenografts were established on 6-week-old female CD1-nu mice by injection of 100 μl 50/50 RPMI/Matrigel solution containing 10^6^ cells of the respective cell line. For xenografts used in therapeutic trials, tumours were established on 4- to 6-week-old female NCr-nu mice (Charles River) by injection of 100 μl 50/50 RPMI/Matrigel solution containing 3 × 10^6^ IMR5 cells (*MYCN* amplified, *ALK* amplified). Mice were randomized to specific treatment when tumours were ≥200 mm^3^. Mice were sacrificed when tumours were 2,000 mm^3^. Tumour mass was inferred using tumour volume using volume to mass of xenograft conversion described in McLean et al.^58^. The *Th-MYCN* mouse model was used to investigate the functional changes of metabolism driven by MYCN. 129×1/SvJ mice transgenic for the *Th-MYCN* construct^10^ were originally obtained from B. Weiss. *Th-MYCN* hemizygous mice were bred and litters randomized to assigned therapy. Mice were genotyped from tail-snip-isolated DNA using quantitative PCR^23^ and only transgene-homozygous mice (*Th-MYCN*^+/+^) were included in these studies. In this model, MYCN expression is targeted to the mouse neural crest under the tyrosine hydroxylase promoter, recapitulating hallmark features of human neuroblastoma. Tumours arise at autochthonous sites in an immunocompetent host with histologic, genomic, and immune similarities to human neuroblastoma^59,60^. Tumours are fully penetrant with onset prior to day 14 in >75% based on histologic audits^61^ and are lethal by 7 weeks.

### Mouse husbandry

Mice were maintained with 12 h of dark daily (18:00 to 06:00). The rodent holding rooms were maintained at a temperature range of 18.9 °C–25.6 °C with an ideal setpoint of 22.2 °C. The humidity was maintained within a range of 30–70% with an ideal setpoint of 50%.

### Metabolite extraction from tissue, tumours and serum

Tissues and tumours were collected from mice in fed state and immediately clamped into liquid nitrogen using Wollenberger clamp. All tissues were stored in −80 °C. Frozen tissues were transferred to 2 ml Eppendorf tubes, which were precooled on dry ice, and then pulverized using Cyromill. The resulting tissue powder was weighed (around 10 mg) and mixed well by vortexing in extraction buffer (40 μl extraction buffer per mg tissue). The extraction solution was neutralized with NH_4_HCO_3_ as above and centrifuged in a microfuge at maximum speed for 30 min at 4 °C. Supernatant was transferred to LC–MS vials for analysis. Blood samples were drawn from mouse tail veins using a microvette and kept on ice. After centrifugation (10 min, benchtop microfuge maximum speed, 4 °C), serum was collected in a 1.5 ml tube and stored at −80 °C. Five microlitres of serum were mixed with 200 μl extraction buffer (40:40:20 acetonitrile: methanol: water with 0.5% formic acid) and neutralized with 15% NH_4_HCO_3_. After centrifugation (30 min, benchtop microfuge maximum speed, 4 °C), supernatant was transferred to LC–MS vials for analysis.

### Metabolite measurement by LC–MS/MS

Metabolomics was performed on the following systems. A quadrupole-orbitrap mass spectrometer (Q Exactive, Thermo Fisher Scientific), operating in positive or negative mode was coupled to hydrophilic interaction chromatography (HILIC) via electrospray ionization^18^. Scans were performed from *m*/*z* 70 to 1,000 at 1 Hz and 140,000 resolution. Liquid chromatography separation was on a XBridge BEH Amide column using a gradient of solvent A (20 mM ammonium acetate, 20 mM ammounium hydroxide in 95:5 water:acetonitrile, pH 9.45) and solvent B (acetonitrile). Flow rate was 150 μl min^−1^. The liquid chromatography gradient was: 0 min, 85% B; 2 min, 85% B; 3 min, 80% B; 5 min, 80% B; 6 min, 75% B; 7 min, 75% B; 8 min, 70% B; 9 min, 70% B; 10 min, 50% B; 12 min, 50% B; 13 min, 25% B; 16 min, 25% B; 18 min, 0% B; 23 min, 0% B; 24 min, 85% B. Autosampler temperature was 5 °C, and injection volume was 5–10 μl. Complementary, primary samples analysed on an Exactive (Thermo Fisher Scientifc) operating in negative ion mode^62^. Liquid chromatography separation was achieved on a Synergy Hydro-RP column (100 mm × 2 mm, 2.5 μm particle size, Phenomenex), using reversed-phase chromatography with the ion pairing agent tributylamine in the aqueous mobile phase to enhance retention and separation. An adaptive scan range was used with an *m*/*z* from 85–1,000. Resolution was 100,000 at 1 Hz. The total run time was 25 min with a flow rate at 200 μl min^−1^. Solvent A is 97:3 water/methanol with 10 mM tributylamine and 15 mM acetic acid; solvent B is methanol. The gradient is 0 min, 0% B; 2.5 min, 0% B; 5 min, 20% B; 7.5 min, 20% B; 13 min, 55% B; 15.5 min, 95% B; 18.5 min, 95% B; 19 min, 0% B; 25 min, 0% B.

### Mass spectrometry analysis

Metabolomics data analysis was performed using ElMaven software (https://github.com/ElucidataInc/ElMaven). For labelling experiments, correction for natural abundance of ^13^C was performed using Accucor (https://github.com/XiaoyangSu/AccuCor).

### Infusion studies and isotope tracing in *Th-MYCN* mice

*Th-MYCN* mice were housed in groups and food was supplied without restriction to guarantee sufficient supply. Mice weights were recorded every day. During experiments mice were freely moving and tissues and serum were analysed following the above-mentioned method. Tumour and inter organ cooperativity in proline, arginine and ornithine biosynthesis was analysed on the whole-body level. The mice were on normal light cycle (06:00–18:00). In vivo infusion was performed on 6- to 7-week-old normal *Th-MYCN* mice pre-catheterized in the right jugular vein and ^13^C metabolite tracers were infused for 2.5–5 h to achieve isotopic pseudo-steady state. The mouse infusion setup included a tether and swivel system, connecting to the button pre-implanted under the back skin of mice. Mice were fasted from 09:00 to 14:00 and infused from 14:00 to 16:30. Tracers were dissolved in saline and infused via the catheter at a constant rate (0.1 μl min^−1^ per g mouse weight) using a Just infusion Syringe Pump. One-hundred millimolar [U-^13^C]glutamine was dissolved and infused for 2.5 h, 40 mM [U-^13^C]arginine was infused for 5 h, 200 mM [U-^13^C]glucose was infused for 5 h, 10 mM [U-^13^C]proline for was infused 5 h and 5 mM [U-^13^C]ornithine was infused for 5 h. At the end of infusion, mice were dissected and tissues were clamped in aluminium foil and stored in liquid nitrogen.

### Intervention study in *Th-MYCN* mice

ArgPro-free diet was purchased from TestDiet Baker (1812426 (5CC7) for CD, 1816284-203 (5WYF) for ProArg-free diet, 1819015-203 (5WZ3) for arginine-free diet and 1816284-203 (5BDL) for proline-free diet). Detailed makeup is given in Supplementary Table 2. The ODC inhibitor, DFMO, was obtained from P. Woster. DFMO was dissolved in drinking water and supplied to mice ad libitum at a dose of 1% in the drinking water. Survival end point: only *Th-MYCN*^+/+^ mice were randomized. DFMO was provided to mothers on day 1 (partially transmitted to pups in breastmilk) and then directly to pups at day 28 of life, post-weaning; diet change was started at day 21 of life, per treatment assignment. Mice were weighed and assessed for tumour growth and symptoms, at least thrice weekly by a single experienced animal technician. Mice were euthanized at pre-defined humane endpoints related to overall health or tumour burden (hunching, immobility, hindlimb paresis, weight loss or respiratory distress). An additional ‘late start’ trial was done with *Th-MYCN*^+/+^ mice enrolled at the time of a palpable progressing abdominal tumour (typically day of life 35–50), randomized to diet and/or DFMO as above, and taken down for tumour mass after 14 days of therapy, sooner if humane endpoints were reached. For metabolomics studies. *Th-MYCN*^+/+^ mice were treated as above, serum was obtained at day 43 (±2 days) and tumours and organs were collected at that time if tumour was present or delayed to the earliest time tumour became palpable. Time to tumour collection depended on the treatment group. In order to ensure homogenous timing of metabolic tumour collection, *Th-MYCN*^+/+^ mice ProArg-free diet plus DFMO had therapy delayed to day 28.

### Intervention study in human xenografts

Mice bearing established IMR5 xenografts at ≥200 mm^3^ were randomized to diets and/or DFMO as above. Mice were weighed and assessed for tumour volume and symptoms, at least thrice weekly. End point: mice were euthanized when tumour volume >2 cm^3^ using calipers measurements and assuming an ellipsoid volume.

### Polyamine quantification

Polyamine concentrations and amino acids in the single-amino-acid trials (Pro-free and Arg-free) were quantified using the AccQ-Tag fluorescence dye (Waters) as described^63^. Derivatives were separated on an Acquity BEH C18 column (150 mm×2.1 mm, 1.7 μm, Waters) by reverse phase UPLC (Acquity H-class UPLC system, Acquity FLR detector, Waters). The column was equilibrated with buffer A (140 mM sodium acetate pH 6.3, 7 mM triethanolamine) at a flow rate of 0.45 ml min^−1^ and heated at 42 °C. Pure acetonitrile served as buffer B. The gradient was produced by the following concentration changes: 1 min 8% B, 7 min 9% B, 7.3 min 15% B, 12.2 min 18% B, 13.1 min 41% B, 15.1 min 80% B, hold for 2.2 min, and return to 8% B in 1.7 min. Chromatograms were recorded and processed with the Empower3 software (Waters). For acetylated polyamines a MS/MS method was used. In brief, a Waters Acquity I-class Plus UPLC system (Binary Solvent Manager, thermostatic Column Manager and FTN Sample Manager) (Waters) coupled to an QTRAP 6500+ (Sciex) mass spectrometer with electrospray ionization (ESI) source was used. Data acquisition was performed with Analyst (Sciex), and data quantification was performed with the SciexOS software suite (Sciex). Chromatography was made on an Acquity HSS T3 column (150 mm × 2.1 mm, 1.7 μm, Waters) kept at 20 °C and a flow rate of 0.3 ml min^−1^. Eluent A consisted of water with 0.1% formic acid and eluent B in acetonitrile with 0.1% formic acid. Gradient elution consisted in changing %B as follows: 0–1 min 0%; 5 min 20%; 5.5–7.5 min 100%, and 8–10 min 0%. The ion source settings were as follow: curtain gas: 30 psi; collision gas: low; ion spray: 4,500 V; source temperature: 500 °C; ion source gas 1: 40 (GS1) and ion source gas 2: 50 (GS2). All compounds were measured in positive electrospray ion mode. To ensure comparability of amino acid levels the signal intensity was scaled based on samples that were in parallel analysed by LC–MS.

### Detection of labelled polyamines in serum and mouse tissues

For fate tracing of ^13^C-labelled amino acids into polyamines an ice-cold extraction solvent consisting of 0.1 M HCl plus 10 μM Norleucine (internal standard) was added as follows: 45 μl for 5 μl of serum and 300 μl for 20–30 mg of mouse tissue. After a 15-min incubation on ice, samples were vortexed and centrifuged at 14,000 rpm for 5 min at 4 °C. The supernatants were then labelled with the fluorescence dye AccQ-Tag (Waters) according to the manufacturer’s protocol, with a modification for serum samples where the final volume was adjusted to 120 μl instead of 500 μl. The method used an I-class UPLC system coupled to a QTRAP 6500+ mass spectrometry system (AB SCIEX) with an electrospray ionization (ESI) source. The derivatives were separated using an Acquity HSS T3 column (100 mm × 2.1 mm, 1.8 µm, Waters) maintained at 40 °C. The mobile phases were: A, 0.1% formic acid in water; and B, 0.1% formic acid in acetonitrile. The mass spectrometer was operated in positive-ion mode with an ion spray voltage of 5,500 V, a source temperature of 550 °C, and GS1 and GS2 set at 70. Data acquisition was performed using Analyst 1.7.2 (AB SCIEX).

### Polyamine concentration uptake inhibitor AMXT

Polyamine concentration were compared to AMXT1501 treated *Th-MYCN* tumours from Gamble et al.^24^.

### Metabolomics and *MYCN* transcriptional activity in 180 cancer cell lines

Metabolomics data for 180 cancer cell lines was obtained from Cherkaoui et al.^64^. Associations between metabolite levels in core metabolic pathways and MYCN transcriptional activity were obtained from https://cancer-metabolomics.azurewebsites.net/page2.

### Gene-expression analysis in neuroblastoma tumours

Gene-expression profiles of 649 neuroblastoma tumours^65^ were obtained from R2 (R2 Genomics Analysis and Visualization Platform; http://r2.amc.nl). Differential expression analysis between MYCN amplification status was performed using the Bioconductor package limma^66^ (v.3.40.6).

### Gene-expression analysis in neuroblastoma cell lines

Expression profiles of 39 neuroblastoma cell lines^67^ were obtained from Gene Expression Omnibus. Differential analysis was performed using the Bioconductor package limma (v.3.40.6) and by comparing MYCN amplification status provided in the study. Data from the CCLE^68^ was taken from the release from the second quarter of 2021 (21Q2).

### RNA and ribosome sequencing

#### Isolation of total RNA, library preparation and sequencing

Total RNA was isolated from the same extracts that were used to obtain mRNA protected fragments (RPFs) (‘Ribo-seq of *Th-MYCN* tumours’). Three volumes of QIAzol (Qiagen, 79306) were added to 80 μl of cell extracts, mixed thoroughly and proceed to RNA purification with Direct-Zol RNA Mini Prep Plus kit. RNA were sent to Genomic Platform (UNIGE) for stranded mRNA libraries preparation. Libraries were sequenced on an Illumina NovaSeq 6000, SR 100 bp, 10 libraries in 1 pool.

#### Ribo-seq of *Th-MYCN* tumours

Mouse tumours were mechanically disrupted in liquid nitrogen and homogenized in a lysis buffer (LB, 50 mM Tris, pH 7.4, 100 mM KCl, 1.5 mM MgCl_2_, 1.0% Triton X-100, 0.5% sodium deoxycholate, 25 U ml^−1^ Turbo DNase I, 1 mM DTT, 100 μg ml^−1^ cycloheximide, and protease inhibitors) 3 ml of LB per 1 g of tissue. To obtain ribosome footprints 0.12 ml of total extracts containing 300 μg of total RNA were treated with RNAse I (Epicentre) (25 U per mg of total RNA), for 45 min at 20 °C with slow agitation. 10 ml SUPERaseIn RNase inhibitor was added to stop nuclease digestion. Monosomes were isolated using S-400 columns. For isolation of RPFs, 3 volumes of QIAzol were added to the S-400 eluate, mixed thoroughly and proceed to RNA purification with Direct-Zol RNA Mini Prep Plus kit.

RPF libraries were prepared as described^69,70^. In brief, RPFs (25–34 nucleotides) were size-selected by electrophoresis using 15% TBE–Urea polyacrylamide gel electrophoresis (PAGE) and two RNA markers, 25-mer (5′-AUGUACACGGAGUCGAGCACCCGCA-3′) and 34-mer (5′-AUGUACACGGAGUCGAGCACCCGCAACGCGAAUG-3′). After dephosphorylation with T4 Polynucleotide Kinase (NEB, M0201S) the adapter Linker-1 (5′-rAppCTGTAGGCACCATCAAT/3ddC/-3′) was ligated to the 3′ end of the RPF using T4 RNA Ligase 2. Ligated products were purified using 10% TBE–Urea PAGE. Ribosomal RNA was subtracted using RiboCop rRNA Depletion Kit V2 H/M/R. The adapter Linker-1 was used for priming reverse transcription with the reverse transcription primer Ni-Ni-9 (5′-AGATCGGAAGAGCGTCGTGTAGGGAAAGAGTGTAGATCTCGGTGGTCGC_5_CACTCA_5_TTCAGACGTGTGCTCTTCCGATCTATTGATGGTGCCTACAG-3′) using ProtoScript II Reverse Transcriptase. Reverse transcription products were purified using 10% TBE–Urea PAGE. The cDNA was circularized with CircLigase II ssDNA Ligase. The final libraries were generated by PCR using forward index primer NI-N-2 (5′-AATGATACGGCGACCACCGAGATCTACAC-3′) and one of the reverse index primers. Amplified libraries were purified using 8% TBE-PAGE and analysed by Qubit and TapeStation. Libraries were sequenced on an Illumina NovaSeq 6000, SR 100 bp, 4 libraries in 1 pool.

#### RNA-seq mapping

Fastq files were adaptor stripped using cutadapt with a minimum length of 15 and a quality cut-off of 2 (parameters: -a CTGTAGGCACCATCAAT –minimum-length = 15 –quality-cutoff = 2). Resulting reads were mapped, using default parameters, with HISAT2^71^, using a GRCm38, release 101 genome and index. Differential expression analysis was performed using DESeq2^72^, using a GRCm38, release 101 genome and index.

#### Ribo-seq mapping

Fastq files were adaptor stripped using cutadapt. Only trimmed reads were retained, with a minimum length of 15 and a quality cut-off of 2 (parameters: -a CTGTAGGCACCATCAAT – trimmed-only –minimum-length = 15 –quality-cutoff = 2). Histograms were produced of ribosome footprint lengths and reads were retained if the trimmed size was 28 or 29 nucleotides. Resulting reads were mapped, using default parameters, with HISAT2^71^ using a GRCm38, release 101 genome and index and were removed if they mapped to rRNA or tRNA according to GRCm38 RepeatMasker definitions from UCSC. A full set of transcripts and coding sequence (CDS) sequences for Ensembl release 101 was then established. Only canonical transcripts (defined by known canonical table, downloaded from UCSC) were retained with their corresponding CDS. Reads were then mapped to the canonical transcriptome with bowtie2 using default parameters.

#### Ribo-seq analysis

The P-site position of each read was predicted by riboWaltz^73^ and confirmed by inspection. Counts were made by aggregating P-sites overlapping with the CDS and P-sites per kilobase million (PPKMs) were then generated through normalizing by CDS length and total counts for the sample. Differential expression and translational efficiency analysis was performed using DESeq2^72^. All metagenes, stalling and ribosome dwelling occupancy (RDO) analyses are carried out on a subset of expressed canonical transcripts which had PPKM values greater than 1 across all samples (10,366 total). Within these, P-site depths per nucleotide were normalized to the mean value in their respective CDS. For metagenes around codon types, the mean of these normalized values is taken for each codon within 90 nucleotides of every instance of that codon. For RDO calculation for a given type of codon, the mean of these normalized values is taken over all instances of that codon, then these are compared using a log_2_-transformed fold change ratio between conditions. To assess relative pausing, P-site depths normalized to the CDS mean were compared at each codon position in the CDS. A value of 1 was added to these normalized depths and a log_2_-transformed fold change ratio was taken pairwise between conditions. To compare effects of different codon ending bases, the resulting values were separated by the ending base of each codon and plotted across their respective positions in the CDS. The relative pausing sum for each ending A, T, G or C is then the sum of these values for every codon containing the respective ending codon across the CDS. The fraction of nucleotides at ending codons were evaluated from extracting the codons for the CDS of each gene using GRCm38, release 101. Pathway level fractions were computed using the average of each gene contained in the pathway.

#### Ribo-seq in cell lines upon pharmacological DHPS or MYCN inhibition

To inhibit hypusination, cells were treated with 6.25 μM of the DHPS inhibitor GC7 (MedChemExpress) for 5 days. For MYCN inhibition, cells were treated with 5 μM MYCi975 (Selleckchem) for 4 days. Western blot analysis was performed to assess the efficacy of the inhibition. Following the respective treatment periods, cells were incubated with 100 μg ml^−1^ cycloheximide (Sigma) for 10 min at 37 °C. Cells were washed once with PBS containing 100 μg ml^−1^ cycloheximide, then trypsinized using a solution containing 100 μg ml^−1^ cycloheximide for 1 min at 37 °C. Cells were pelleted by centrifugation and washed with cold PBS containing 100 μg ml^−1^ cycloheximide. IMR5 cells were disrupted in a lysis buffer (20 mM Tris, pH 7.4, 140 mM KCl, 5 mM MgCl_2_, 1.0% Triton X-100, 1 mg ml^−1^ heparin, 25 U ml^−1^ Turbo DNase I (Roche, 04716728001), 1 mM DTT, 100 μg ml^−1^ cycloheximide (Sigma, C7698) and protease inhibitors (Roche, 04693132001). To obtain ribosome footprints 80 μl of lysates containing 150 g of total RNAs were treated with RNAse I (Ambion, AM2295) (250 U per mg of total RNA), for 60 min at 20°С with gentle agitation. Four microlitres SUPERaseIn RNase inhibitor (Ambion, AM2694) was added to stop nuclease digestion. Monosomes were isolated using S-400 columns (Cytiva, 27514001). Samples were then processed using Ribo-seq as described above.

#### Ribo-seq analysis of genetic inhibition of hypusination

Ribo-seq data from lymphoma cells with shRNAs targeting Renilla (sh-contl), *Eif5a* or *Dhps* were taken from Nakanishi et al.^29^. Raw data were downloaded from Gene Expression Omnibus (GEO) accession GSE190670 and were processed using Ribo-seq analysis as described above.

#### Ribo-seq analysis of genetic induction of MYC

Reprocessed and reanalysed data were from Elkon et al.^74^. Raw data were downloaded from GEO GSE66927 and were processed using Ribo-seq analysis as described above.

#### Ribo-seq analysis of genetic induction of *MYCN* amplified cell lines

Reprocessed and reanalysed data from Volegova et al.^75^. Raw data were downloaded from GEO accession GSE261760 and were processed using Ribo-seq analysis as described above.

### In vitro experiments in neuroblastoma cell lines

#### Cell growth analysis

IMR5 cells were cultured in RPMI medium supplemented with 10% dialysed FBS (Gibco) and seeded into 384-well plates. Stock solutions of arginine, proline, DFMO and GC7 were dispensed into the 384-well plates using the Echo 650 (Beckmann Coulter) liquid handler in a concentration-dependent manner. The impact of varying proline and arginine concentration was assessed using RPMI free of arginine and proline and supplemented with 0%, 1%, 5%, 30%, 50% and 100% of standard RPMI levels of proline (174 μM) and arginine (1,149 μM). For polyamine supplementation, putrescine, spermidine or 1-methyl-spermidine (BenchChem), were supplemented in the respective concentrations as described above along with 1 mM aminoguanidine (Sigma-Aldrich). Additional rescue experiments were performed in different neuroblastoma cell backgrounds (SHEP, SKNBE2 and SHSY5Y). For quantification cells were then stained with 10 μM Hoechst 33342 (Invitrogen) and 1 μg ml^−1^ propidium iodide (Invitrogen) for 15 min at 37 °C/5% CO_2_. Fluorescent signals were captured and analysed at different time points using the Perkin-Elmer Operetta system to generate growth curve.

#### shRNA-mediated gene knockdown

Lentiviral particles were produced in HEK293 cells by co-transfection of lentiviral packaging plasmids pCMV-VSV-G and pPAX2 (Addgene) with pRSIT-U6Tet-shTarget-PGK-TetRep-2A-TagGFP2-2A-Puro (Cellecta) expressing the desired shRNAs using 25 kDa linear polyethylenimine (Polysciences). Viral supernatants were collected 48 h post-transfection. IMR5 cells were transduced with viral supernatants containing 4 μg ml^−1^ polybrene (Sigma) for 48 h. Cells were selected with 2 μg ml^−1^ puromycin (Gibco) 48 h post-transduction. 50 ng ml^−1^ doxycycline (Sigma) was used for at least 24 h to induce shRNA expression. The construct targets are reported in Supplementary Table 3.

#### Cell cycle analysis

Cells were seeded into 12-well plates at the density of 2 × 10^4^ cells per ml and induced with 50 ng ml^−1^ doxycycline for 5 days. Cells were collected and incubated for 1 h in growth medium containing 10 μg ml^−1^ Hoechst 33342(Invitrogen). The stained cells were then analysed by flow cytometry and cell cycle distribution was quantified using the Modfit software.

#### Puromycin incorporation

IMR5 cells were treated with 500 μM DFMO in 20% ProArg RPMI medium supplemented with 10% dialysed FBS for 5 days. Cells in log growth phase (< 80% confluence) were labelled with 1 μM puromycin at 37 °C for 1 h, then the medium was replaced by Versene (Gibco) and 5 μM cycloheximide (Sigma) to inhibit protein translation. For western blot analysis lysates were separated electrophoretically and transferred to PVDF membranes (Bio-Rad), blocked with 5% non-fat milk in TBS-T, and detected with a mouse monoclonal puromycin antibody (Millipore, 1:10,000) at 4 °C overnight, followed by horseradish peroxidase-conjugated goat anti-mouse secondary (Proteintech, 1:10,000) for 1 h at room temperature. Protein bands were visualized using enhanced chemiluminescence (ECL) reagent (Bio-Rad) and imaged on a ChemiDoc system (Bio-Rad). For flow cytometry analysis, cells were fixed in 4% formalin at room temperature for 10 min and permeabilized with 0.1% Triton X-100 for 10 min. Cells were stained with PE anti-puromycin (BioLegend) for 30 min at 4 °C and analysed on a flow cytometer (Sony).

#### Western blotting of neuroblastoma cell line lysates

After cell lysis samples were transferred to PVDF membranes as described above. Antibodies used were used in the following concentrations: ODC1 (1:1,000, Abcam), MYCN (1:1,000, Santa Cruz), eIF5a (1:2,000, BD), eIF5A anti-hypusine (1:2,000, Merck Millipore), CENPR (1:1,000, Proteintech), KIF2C (1:1,000, Proteintech) and horseradish peroxidase-conjugated anti-GAPDH (1:10,000, Proteintech).

#### RT–qPCR

Total RNA from cells was isolated using Trizol (Invitrogen) according to the manufacturer’s instructions. One microgram of total RNA was used to generate cDNA adapted for quantitative PCR with reverse transcription (RT–qPCR) (TaKaRa Bio). Real-time PCR was carried out using a Quant Studio 7 Pro Real-Time PCR machine (Applied Biosystems) and GoTaq qPCR Master Mix (Promega). Fold change of gene expression was calculated by the 2^−∆∆*C*t^ formula using GAPDH as an endogenous reference. The list of primers for gene-expression analysis using RT–qPCR is reported in Supplementary Table 3.

#### In vitro translation

Template creation: DNA constructs NheI_IRES_HA_EcoRI_xxx_BamHI and nLuc_Myc_EcoRV were synthesized by Gene Script and cloned with NheI and EcoRV sites into pcDNA3.1+ vector. Triplet sequences were inserted between HA and nLuc using EcoRI and BamHI sites: 7× CCG-Pro and 7× CCA-Pro. DNA plasmids were linearized with NotI HF (NEB). In vitro T7 transcription with linear plasmids was performed with mMESSAGE mMACHINE Kit (Invitrogen, AM1344). For the cell lysate preparation IMR5 cells were seeded in 15 cm dish at a density of 1 × 10^6^ per dish and cultured for 5 days. Cells were then collected and centrifuged at 1,000*g* for 5 min at 4 °C. Cell pellets (100 mg) were lysed in 1 volume (100 μl) of lysis buffer (LB, 30 mM HEPES/KOH (pH 7.6), 150 mM potassium acetate, 3.9 mM magnesium actetate, 4 mM DTT, 1% Triton X-100, 10% glycerol and protease inhibitor (Roche, 04693132001). Four microlitres SUPERaseIn RNase inhibitor (Ambion, was added. Cell debris was removed by centrifugation at 20,000*g* for 10 min at 4 °C. RNA and protein concentration were quantified in the cell lysate. Forty-microlitre aliquots of the cell lysates were snap frozen in liquid nitrogen and stored at −80 °C. Six microlitres were used for the in vitro translation reaction. In vitro translation was performed in a translational mix (15 mM HEPES/KOH (pH 7.6), 75 mM potassium acetate, 2 mM magnesium actetate, 1.75 mM ATP, 0.4 mM GTP, 50 μM complete amino acid mix (Promega, L4461), 20 mM creatine phosphate, 0.3 mg ml^−1^ creatine kinase, 500 ng RNA) for 3 h at 35 °C. Spermidine was added in a final concentration 1.5 mM, if indicated. Translation reaction product was detected with Nano-Glo Luciferase Assay System (Promega, N1110).

#### Immunoblots of *Th-MYCN* tumours

Collected *Th-MYCN* tumours were clamped and flash-frozen in liquid nitrogen. After this mechanical dissociation, crude protein extraction was obtained by lysis with CHAPS buffer (10 mM HEPES, 150 mM NaCl, 2% CHAPS) with fresh protease inhibitor and phosphatase inhibitor. This protein lysate (25 micrograms) was electrophoresed through a 5–10% Tris–glycine gel and immunoblotted using antibodies to MYCN (1:500, Cell Signaling), GAPDH (1:3,000, Cell Signaling Technologies) and eIF5A anti-hypusine (1:2,000, Millipore Sigma).

#### Isoelectric focusing blots

Crude protein extracts obtained as described above were electrophoresed through a slab isoelectric focusing gel (pH 3–7, Invitrogen Novex EC66452) with freshly made cathode and anode buffers (Novex). The gel was transferred to a PVDF membrane and transferred using the iBlot transfer unit prior to blocking in buffer according to manufacturer’s recommendations for iBind. The iBind was then assembled with a probe against eIF5A (1:3,000, BD Laboratories) and incubated for at least 2.5 h before developing.

#### Histology

Collected *Th-MYCN* tumours were preserved in 10% formalin and embedded in paraffin blocks. Slides were cut and then stained with H&E. These slides were reviewed by a pathologist blinded to the treatment groups, and tumours were scored according to: (1) differentiation status; (2) neuropil presence or absence and relative abundance; and (3) evidence of global or localized necrosis. Slides were then scanned and re-reviewed by the same pathologist.

#### Immunohistochemistry in *Th-MYCN* tumours

Slides of formalin fixed, paraffin embedded tumours were stained on a Bond Max (MYCN, Ki67) automated staining system (Leica Microsystems). The Bond Refine staining kit was used for MYCN and Ki67. For MYCN (1:100, Abcam) and Ki67 (1:200, Abcam), the standard protocol was followed with the exception of the primary antibody incubation which was extended to 1 h at room temperature. Antigen retrieval was performed with E2 (Leica Microsystems) retrieval solution for 20 min. After staining, all slides were rinsed, dehydrated through a series of ascending concentrations of ethanol and xylene, then cover-slipped. Stained slides were then digitally scanned at 20× magnification on an Aperio CS-O or AT2 slide scanner (Leica Biosystems) and reviewed by a pathologist blinded to the treatment groups.

#### LC–MS/MS analysis of tRNA ribonucleosides

Tumours were collected and pulverized as described above. From the resulting tissue powder total RNA was extracted using TRIzol according to the manufacturer’s instructions (Invitrogen) followed by tRNA isolation by gel extraction from denaturing 8 M urea, 8% polyacrylamide gels. Gel-extracted tRNA (560 ng) was enzymatically digested with 0.8 U nuclease P1 from *Penicillium citrinum* (Sigma, N8630) and 80 U GENIUS nuclease (Santa Cruz Biotech, sc-391121b) in 10 mM ammonium acetate (pH 6.0), 1.0 mM magnesium chloride at 40 °C for 70 min. Hydrolysed ribonucleotides were dephosphorylated at 37 °C for 70 min by 0.4 U snake venom phosphodiesterase (Sigma, P3243) and 0.16 U alkaline phosphatase from *Escherichia coli* (Sigma, P5931) after adjusting the pH with ammonium bicarbonate to a final concentration of 50 mM. The hydrolysates were mixed with 3 volumes of acetonitrile and centrifuged (16,000*g*, 40 min, 4 °C). The supernatants were lyophilized and dissolved in H_2_O for LC–MS/MS analysis. Four biological replicates for each condition (CD, CD plus DFMO, ProArg-free, ProArg-free plus DFMO) were measured, each in two technical replicates. Nucleosides were separated via reversed-phase chromatography using a Vanqush Neo UHPLC system (Thermo Fisher Scientific) and an Acquity nanoEase M/Z Peptide BEH C18 Column (130 Å, 1.7 μm, 300 μm × 150 mm; Waters, 186009259) and analysed on an Orbitrap Exploris 480 mass spectrometer (Thermo Fisher Scientific) operating in a positive-ion mode at a resolution of 45,000, the AGC target value set to 1.0 × 10^6^ and the fill time to 50 ms. Full MS spectra (*m*/*z* 244–576) and Top7 ddMS^2^ spectra with a nominal collision energy of 85% were recorded. Quantitative analysis of LC–MS/MS data was performed using ElMaven software (https://github.com/ElucidataInc/ElMaven) and the identities of quantified ribonucleosides were verified by their specific fragmentation patterns in MS2 and by predetermined chromatographic elution orders of structural isomers^76,77^. Normalization was performed to the mean intensity of 32 nucleosides that were detected in all measurements. For nucleosides that showed a marked difference in intensity between the two technical replicates due to degradation, the second technical replicate was excluded.

### Proteomics

#### Total proteome sample preparation

Tissues were disrupted by grinding in frozen state and lysed in lysis buffer (20 mM HEPES pH 7.2, 2% SDS). Proteins extracts were diluted 1:1 with 2× SDS buffer (10% SDS, 100 mM Tris pH 8.5), boiled for 10 min at 95 °C, reduced with 5 mM (final) TCEP for 15 min at 55 °C, and alkylated with 20 mM (final) CAA for 30 min at room temperature. Proteins were acidified by addition of 3% (final) perchloric acid, followed by addition of seven volumes of binding buffer (90% methanol, 100 mM TEAB). Samples were loaded on S-trap columns and processed on a Resolvex A-200 positive pressure unit (Tecan). Samples were washed 1× with binding buffer, 3× with 50% methanol/50% CHCl_3_ and 2× with binding buffer. Digestion buffer (150 μl TEAB 50 mM) containing trypsin 1:10 (wt:wt, enzyme:protein) and Lys-C mix 1:50 (wt:wt, enzyme:protein) was added and incubated for 1 h at 37 °C. One-hundred microlitres of digest buffer was added, and incubated overnight. Peptides were eluted with 80 μl 0.2% aqueous formic acid followed by 80 μl of 50% acetonitrile containing 0.2% formic acid. Peptides were diluted 1:1 with STOP buffer (PreOmics) and purified over iST positive pressure plates (PreOmics) according to the manufacturer’s instructions.

#### Nanoflow LC–MS/MS measurements for proteomes

Peptides were separated on an Aurora (Gen3) 25 cm, 75 μm internal diameter column packed with C18 beads (1.7 μm) (IonOpticks) using a Vanquish Neo (Thermo Fisher Scientific) UHPLC. Peptide separation was performed using a 90-min gradient of 2–17% solvent B (0.1% formic acid in acetonitrile) for 56 min, 17–25% solvent B for 21 min, 25–35% solvent B for 13 min, using a constant flow rate of 400 nl min^−1^. Column temperature was controlled at 50 °C. Mass spectrometry data were acquired with a timsTOF HT (Bruker Daltonics) in diaPASEF mode. Mass spectrometry data were collected over a 100–1,700 *m*/*z* range. During each MS/MS data collection each PASEF cycle was 1.8 s. Ion mobility was calibrated using 3 Agilent ESI-L Tuning Mix ions: 622.0289, 922.0097 and 1221.9906. For diaPASEF we used the long-gradient method which included 16 diaPASEF scans with two 25 Da windows per ramp, mass range 400.0–1,201.0 Da and mobility range 1.43–0.6 1/*K*_0_. The collision energy was decreased linearly from 59 eV at 1/*K*_0_ = 1.6 to 20 eV at 1/*K*_0_ = 0.6 V cm^−2^. Both accumulation time and PASEF ramp time was set to 100 ms.

#### Mass spectrometry data quantification

Raw mass spectrometry data were analysed with Spectronaut (v.17.1) in directDIA mode with standard settings. Database search included the mouse Uniprot FASTA database.

#### Proteomics analyses

Protein intensity values were normalized by log_2_ transformation and proteins with less than 70% of valid values in at least one group were filtered out. The remaining missing values were imputed using the mixed imputation approach^78^. In brief, missing values in samples belonging to the same group were imputed with *k*-nearest neighbours if there is at least 60% of valid values in that group, for that protein. The remaining missing values are imputed with the MinProb method (random draws from a Gaussian distribution; width = 0.2 and downshift = 1.8).

#### Circulating turnover flux

To measure the circulating turnover flux of a metabolite, we infused [U-^13^C]labelled form of the respective metabolite via the jugular venous catheter. At pseudo-steady state, the fraction of the labelled of mass isotopomer form [*M* + i] of the nutrient in serum is measured as ${L}_{[M+{i}]}$, such that i is from 0 to C and C is the total number of carbons in the metabolite. The circulatory turnover flux *F*_circ_ is defined as previously^79^:

1 $${F}_{{\rm{circ}}}=R\times \frac{1-{L}_{[M+C]}}{{L}_{[M+C]}}$$

where *R* is the infusion rate of the labelled tracer. Since the turnover flux is a pseudo-steady state measurement, for minimally perturbative tracer infusions, production flux is approximately equal to consumption flux of the metabolite and thus *F*_circ_ reflects both the circulating production and consumption fluxes of the infused metabolite in units of nmolC min^−1^ g^−1^.

The carbon atom circulatory turnover flux ${F}_{{\rm{circ}}}^{{\rm{atom}}}$ of the nutrient is calculated using

2 $${F}_{{\rm{circ}}}^{{\rm{atom}}}=C\times R\times \frac{1-L}{L}$$

where *L* is the fraction of labelled carbon atoms in the nutrient:

3 $$L=\frac{{\sum }_{i=0}^{C}i\times {L}_{[M+i]}}{C}$$

${F}_{{\rm{circ}}}$ measures the turnover of the whole carbon skeleton of the molecule, whereas ${F}_{{\rm{circ}}}^{{\rm{atom}}}$ measures the turnover of the carbon atoms in the molecule.

##### Normalized metabolite labelling

When a [U-^13^C]-labelled tracer *X* is infused, the normalized labelling of downstream metabolite *Y* is defined as ${L}_{Y\leftarrow X}=\frac{{L}_{Y}}{{L}_{X}}$, where *L*_*X*_ and *L*_*Y*_ are the fraction of labelled carbon atoms for metabolite *X* and *Y* defined in equation (3).

##### Fractional contribution to tissue metabolites

Direct fractional contribution of each metabolite to other metabolites in a tissue is calculated by setting up the follow set of linear equations:

4 $$M\cdot \left(\begin{array}{c}\begin{array}{c}{f}_{k\leftarrow {\rm{proline}}}\\ {f}_{k\leftarrow {\rm{arginine}}}\\ \vdots \end{array}\\ {f}_{k\leftarrow {\rm{glutamine}}}\end{array}\right)=\left(\begin{array}{c}\begin{array}{c}{L}_{k\leftarrow {\rm{proline}}}\\ {L}_{k\leftarrow {\rm{arginine}}}\\ \vdots \end{array}\\ {L}_{k\leftarrow {\rm{glutamine}}}\end{array}\right)$$

Where ${f}_{k\leftarrow i}$ is the fraction of *k* derived directly from *i*, *M* is the circulating metabolite interconversion matrix and is taken such that entry (*X*,*Y*) represents ${L}_{Y\leftarrow X}$. Direct contributions to tissue were then calculated by performing an optimization procedure conditional on non-negative values by finding min ||*M*, *f* – *L*|| with respect to *f* such that *f* > 0. Standard error was estimated using a bootstrapping method (*n* = 100 simulations) by selecting values for *M* and *L* from normal distributions with means and standard deviations equal to calculated values for those parameters based on measured data.

##### Circulating metabolites flux

The procedure for calculating fluxes between circulating nutrients has been previously described thoroughly^79^. The input data for this calculation are the inter-labelling matrix *M* that reflects the extent to which infusion of any nutrient *i* (of *n* total nutrients of interest) labels every other circulating nutrient *j* and the carbon atom circulatory turnover flux for each circulating nutrient ${F}_{{\rm{circ}}}^{{\rm{atom}}}$*.* The procedure first uses *M* to calculate the direct contributions to each nutrient *i* from all other circulating nutrients *j*, creating a new *n* × *n* matrix *N* whose entries *N*_*ij*_ reflect the direct contributions of circulating nutrient *j* to circulating nutrient *i*. It then utilizes the matrix *N* and ${F}_{{\rm{circ}}}^{{\rm{atom}}}$ to calculate the direct contributing fluxes from any circulating nutrient to any other circulating nutrient, resulting in a complete determination of the inter-converting fluxes in units of nmolC min^−1^ g^−1^ between circulating nutrients. Fluxes were computed using Matlab (v.R2019a). The network was visualized using Cytoscape (v2.9.0).

##### Multi-omics GSEA

All omics were analysed and visualized in R (Statistical Computing, v.4.1.0). Gene set enrichment was performed using fgsea (https://bioconductor.org/packages/release/bioc/html/fgsea.html) using as input gene or protein list rank by relative changes (log_2_-transformed fold change of comparison). Gene sets were taken from the Mouse MSigDB Collections using the gene sets MH (Hallmark) and the M2 (canonical pathways) using the Reactome subset^80^. We used 1,000 permutations of the gene-level values to calculate NES and statistical significance.

##### Neuroblastoma *MYCN-*driven regulatory circuits transcript and protein levels

The super enhancer core regulatory circuitry has been described by Durbin et al.^40^ and modified by Decaesteker et al.^54^. The retino-sympathetic and adrenergic circuit has been described by Zimmerman et al.^81^.

##### Deconvolution of cell types from bulk RNA-seq

To determine cell-type abundance a deconvolution approach was used leveraging the bulk gene-expression profiles of the *Th-MYCN* tumours for the four treatment groups: CD, ProArg-free diet, CD plus DFMO and ProArg-free diet plus DFMO. We used the CIBERSORTx^82^, a machine learning method to infer cell-type proportions without physical cell isolation based on the creation of a signature matrix of cell types identified in *Th-MYCN* tumour model in published single-cell RNA-sequencing data by Costa et al.^83^. Using this high-resolution single-cell annotation of the tumour microenvironment in *Th-MYCN* mice the digital cytometry tool was run on bulk tumour expression to extract cell-type abundances and dissect the effect of treatment on the tumour microenvironment.

### Statistics and reproducibility

The number of human and mouse samples is recorded in the figure captions. For human tumour measurements, *n* represents the number of patients. For mouse experiments, *n* represents the number of mice. *P* values were computed using an unpaired two-sided Welch’s *t*-test using the Welch–Satterthwaite equation (not assuming equal variances) unless specified otherwise. Regression between adenosine-ending codon and protein levels were calculated with the R function stat_cor (package ggpubr) to compute Pearson’s *r* and geom_smooth (package ggplot2) using ‘linear model’ to display the regression line. Statistics were performed using R (v.4.1.0).

#### Metabolomics

A two-tailed unpaired Welch’s *t*-test was used to calculate *P* values. Metabolomics data were corrected for multiple comparisons using the Benjamini–Hochberg method, with a FDR cut-off of 0.05 used to determine statistical significance.

#### Transcriptomics

*P* values were corrected for multiple hypothesis testing using the Benjamini–Hochberg method, with a FDR cut-off of 0.05 used to determine statistical significance.

#### Proteomics

Two-sided Student’s *t*-tests were calculated with a permutation-based FDR cut-off of 0.05 and *s*_0_ = 1 if not otherwise declared.

#### tRNA mass spectrometry

A two-tailed unpaired Welch’s *t*-test was used to calculate *P* values. Modifications were corrected for multiple comparisons using the Benjamini–Hochberg method, with an FDR cut-off of 0.05 used to determine statistical significance.

#### Mouse survival analyses

Comparisons of outcome between groups were performed by a two-sided log-rank test, tumour-related death is counted as an event, with mice censored at the time of death without tumour or necropsy. Survival was statistically assessed according to the method of Kaplan and Meier^84^ according to Peto and Peto^85^.

#### Reproducibility

Representative results, such as blots and H&E staining, were independently validated in at least two independent experiments yielding similar results. This include Fig. 6g,h, Extended Data Figs. 8d,e,n and 9i and Supplementary Figs. 2b, 3b, 4f and 7b.

#### Ethics statement

Animal studies followed protocols approved by the Princeton University and Children’s Hospital of Philadelphia Institutional Animal Care and Use Committees. Patient samples obtained from the COG (Neuroblastoma Biology Committee) underwent review and approval through a Cancer Therapy Evaluation Program overseen process: application ANBL16B2 Q, principal investigator J.D.R.

### Reporting summary

Further information on research design is available in the Nature Portfolio Reporting Summary linked to this article.

## Online content

Any methods, additional references, Nature Portfolio reporting summaries, source data, extended data, supplementary information, acknowledgements, peer review information; details of author contributions and competing interests; and statements of data and code availability are available at 10.1038/s41586-025-09564-0.

## Supplementary information

Supplementary Information This file contains Supplementary Figs. 1–7 and Supplementary Tables 1–3

Reporting Summary

Supplementary Data Uncropped blots used in the main figures and extended data figures.

Supplementary Data Source data for Supplementary Figs. 1–7

## Source data

Source Data Fig. 1

Source Data Fig. 2

Source Data Fig. 3

Source Data Fig. 4

Source Data Fig. 5

Source Data Fig. 6

Source Data Extended Data Fig. 1

Source Data Extended Data Fig. 2

Source Data Extended Data Fig. 3

Source Data Extended Data Fig. 4

Source Data Extended Data Fig. 5

Source Data Extended Data Fig. 6

Source Data Extended Data Fig. 7

Source Data Extended Data Fig. 8

Source Data Extended Data Fig. 9

Source Data Extended Data Fig. 10

## Data Availability

The RNA-seq and Ribo-seq data are accessible under the GEO accession GSE244378. The mass spectrometry proteomics data were deposited to the ProteomeXchange Consortium via the PRIDE partner repository with the dataset identifier PXD047396. Any additional data reported in this manuscript are available from the corresponding author upon request. Source data are provided with this paper.
