# Supplementary material for: Reprogramming neuroblastoma by diet-enhanced polyamine depletion
Source: Nature. 2025 Sep 24;646(8085):707–15. doi: 10.1038/s41586-025-09564-0 (PMC12527938; doi:10.1038/s41586-025-09564-0)
Supplement: Supplementary file 1 — This file contains Supplementary Figs. 1–7 and Supplementary Tables 1–3 [file 41586_2025_9564_MOESM1_ESM.pdf]

nature portfolio

https://doi.org/10.1038/s41586-025-09564-0

Supplementary information

Reprogramming neuroblastoma by diet-enhanced polyamine depletion

In the format provided by the authors and unedited

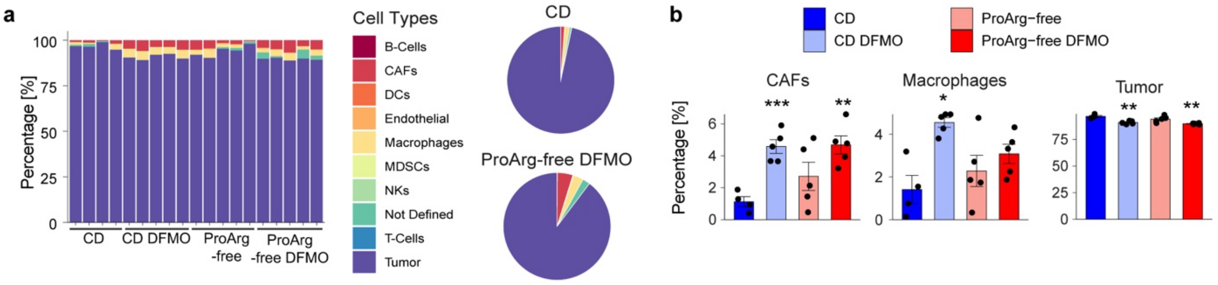

Supplementary Fig. 1: Combined ProArg-free DFMO treatment increases the stromal compartment fraction

a) Proportion of cell types in each tumor under the respective treatment as derived from deconvolution of bulk gene expression from the TH-*MYCN* mice (RNA-seq) (left). The average proportion of stromal cells in ProArg-free DFMO treated tumors is increased, as compared to CD tumors (right).

b) Percentage of most changing cell types according to treatment groups and cell type identity: CAFs, macrophages and tumor cells.

For b: *P < 0.05, **P < 0.01, ***P < 0.001, ****P < 0.0001, two-tailed t-test. Mean ± s.e.m..

For a-b: CD DFMO, ProArg-free and ProArg-free DFMO n = 5; CD n = 4.

Abbreviations: CAFs, cancer associated fibroblast; DCs, Dendritic cells; MDSCs, Myeloid-derived suppressor cells; NKs, natural killer cells.

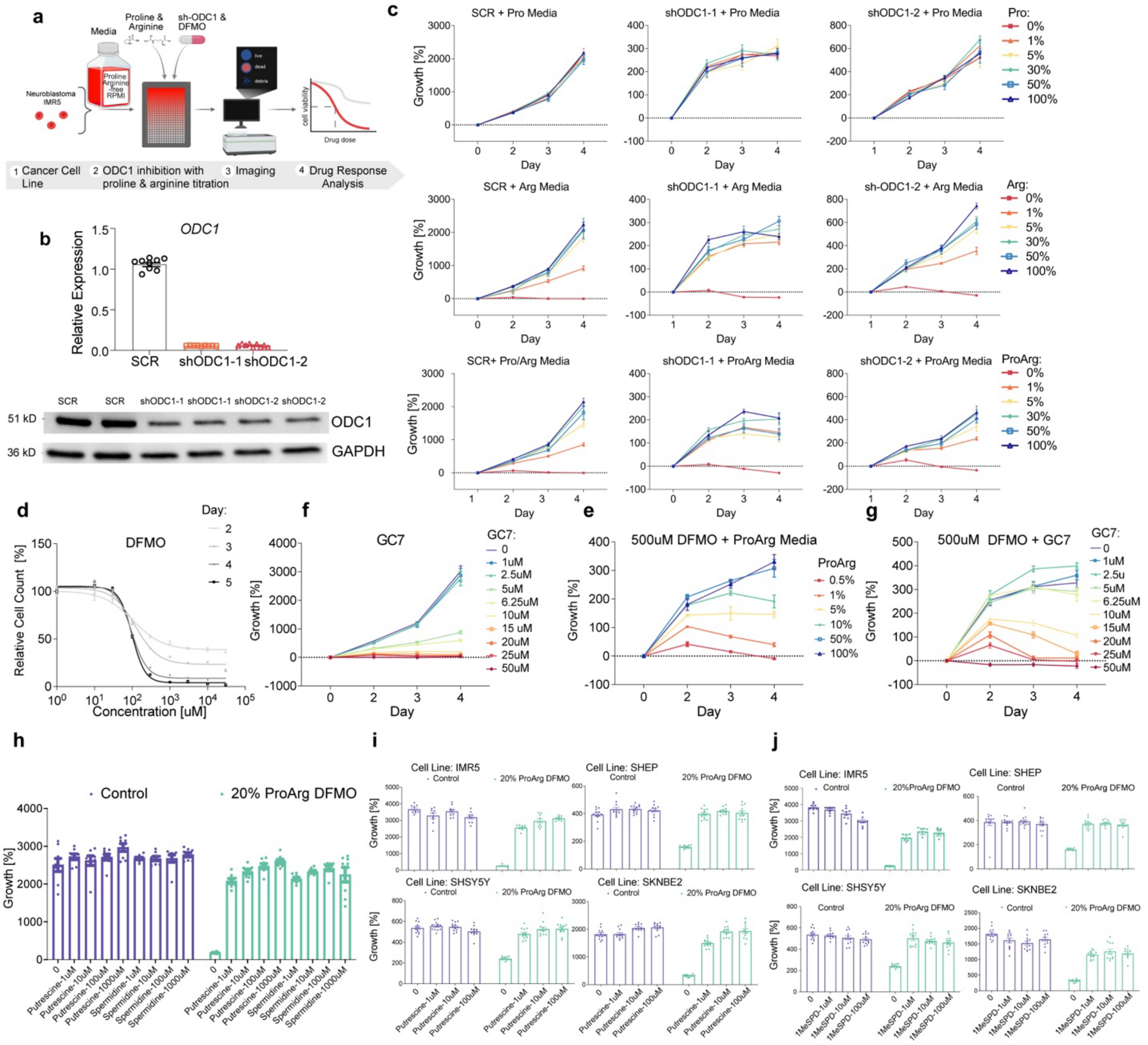

Supplementary Fig. 2: *In vitro* phenotyping of polyamine metabolism in neuroblastoma cell lines

a) Schematic of automated imaging-based quantification of neuroblastoma cell line growth (live cells) across RPMI-based media conditions in combination with either genetic models (sh-RNA expression modulation) or drug treatment. Analyses are performed in the IMR5 *MYCN*-amplified cell line background.

b) Relative expression of ODC1 mRNA upon knock down (KD) by two independent short hairpins (sh) targeting ODC1 or scrambled control (SCR) and related western blot of ODC1 protein levels upon KD confirms reduced protein levels with two independent sh-ODC1.

c) Cell growth over time upon induction of sh-scramble (SCR) or sh-ODC1 in combination with titration of proline and arginine levels in a RPMI media base. Percent values are relative to RPMI concentrations. Mean ± s.e.m..*n* = 4.

d) DFMO dose response in relation to time in the IMR5 neuroblastoma cell line. *n* = 6.

e) IMR5 cell growth upon titration of ProArg media to enhance growth inhibition upon combination with DFMO at a dose of 500 uM. Mean ± s.e.m.. *n* = 4.

f) Impact of GC7, a pharmacological inhibitor of the polyamine dependent enzyme DHPS on IMR5 cell growth. Mean ± s.e.m.. *n* = 4.

g) Cell growth under varying concentrations of GC7 in combination with DFMO at 500 uM in the IMR5 cell line. Mean ± s.e.m.. *n* = 4.

h) Cell growth after 20% ProArg media depletion combined with DFMO at 500uM is rescued by supplementation of different polyamine species (putrescine and spermidine). Aminoguanidine in the media is used to prevent the formation of cytotoxic byproducts from spermidine. Mean ± s.e.m.. *n* = 8.

i) Supplementation of putrescine or j) 1-methyl-spermidine (1MeSPD) upon intracellular polyamine depletion via proline and arginine depletion and DFMO treatment in different neuroblastoma cell lines. Cell growth in control and treatment with 500uM DFMO with 20%ProArg media in IMR5 *n* = 9, SHSY5Y, SHEP and SKNBE2 cell lines *n* = 12. Mean ± s.e.m..Abbreviations: sh, short hairpin; ODC1, ornithine decarboxylase 1; KD, knock down; SCR, scramble; Pro, proline; Arg, arginine; DFMO, difluoromethylornithine.

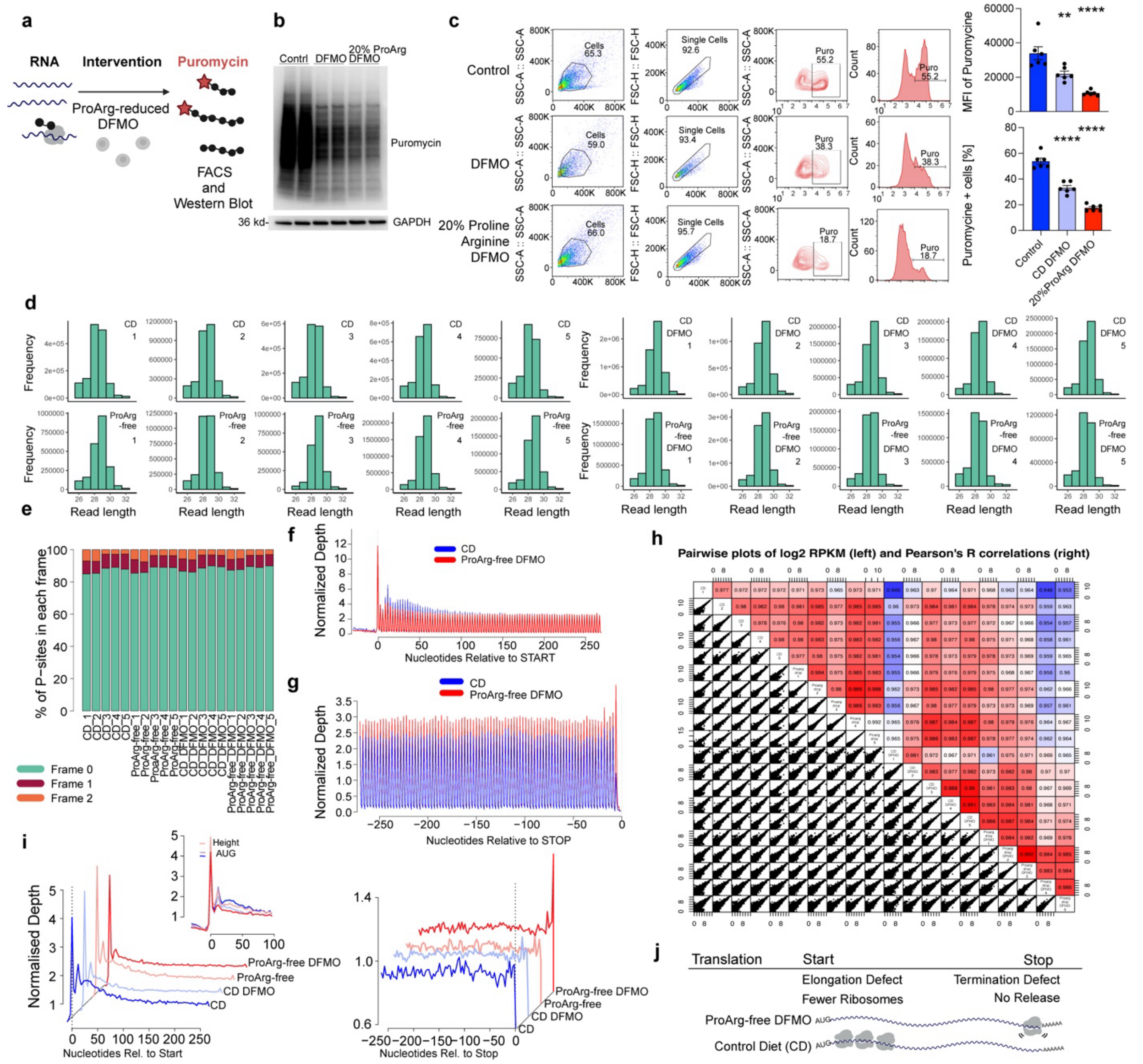

Supplementary Fig. 3: Evaluation of translation using puromycin incorporation and Ribo-Seq quality control

a) Changes in translation in the IMR5 neuroblastoma cell line were quantified by incorporation of puromycin in newly synthesized peptides upon pulse labeling with 1 uM at 37°C for 1 hour. Analysis was performed upon treatment with 500 uM DFMO for 5 days in combination with full RPMI media or reduced proline and arginine concentrations (20% ProArg DFMO).

b) Semi-quantitative assessment of translation rates by western blot based detection of puromycin incorporation into proteins using a α-puromycin antibody. Combining DFMO with proline and arginine depletion further reduces global protein synthesis. Experiment repeated two times on different days. Representative result.

c) Flow cytometry-based quantification of puromycin incorporation in proteins of IMR cells. Quantitative readouts are given as mean fluorescence intensity (MFI) or percentage of positive cells upon staining with puromycin specific antibodies highlight a further reduction of protein biosynthesis in the combined treatment. Mean ± s.e.m.. *$P < 0.05$, **$P < 0.01$, ***$P < 0.001$, ****$P < 0.0001$, two-tailed t-test. $n = 6$ per group.

d) Read length frequency distribution of ribosome footprints of each measured sample showing no group specific differences.

e) Periodicity by frame for each measured sample showing high frame 0 concordance across the cohort.

f-g) Nucleotide resolution ribosome occupancy indicating high periodicity of translation at the transcript 5' and 3' comparing CD and ProArg-free DFMO samples. Mean.

h) Pairwise ribosome profile correlation between samples.
i) Normalized ribosome density across transcripts. Combining a proline and arginine-free diet with DFMO affects the global ribosome distribution at initiation/early elongation (left + insert) and causes a termination defect (right). *n* = 5. j) Schematic of translation defect. Abbreviations: Ribo-Seq, Ribosome Profiling; CD, control diet; ProArg-free, proline and arginine-depleted diet; DFMO, difluoromethylornithine.

For d-h: *n* = 5. Abbreviations: CD, control diet; ProArg-free, proline arginine free diet; DFMO, difluoromethylornithine.

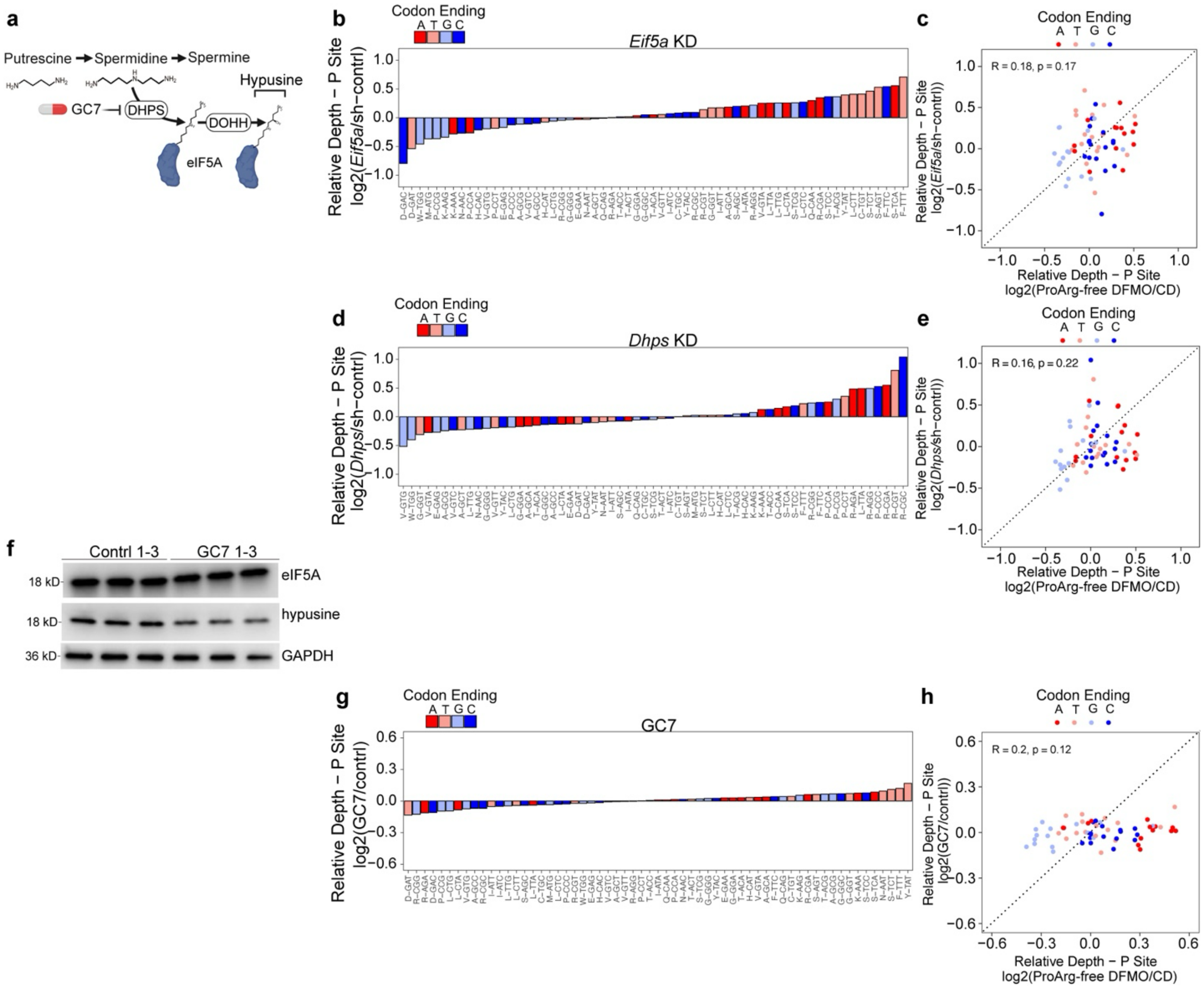

Supplementary Fig. 4: Hypusination inhibition does not cause polyamine depletion codon-specific translation defects

a) Schematic of the role of DHPS in eIF5A hypusination and the tool-compound GC7 blocking eIF5A hypusination.

b) *Eif5a* KD pausing at the ribosomal P site does not show pausing at adenosine-ending codons relative to sh-control (left).

c) Direct comparison of translation phenotype in ProArg-free DFMO to *Eif5a* KD shows no correlation in relative codon pausing.

d) Loss of eIF5a hypusination by *Dhps* KD is not characterized by ribosome pausing at adenosine-ending codons given as relative depth at ribosomal P site.

e) Direct comparison of translation phenotype in ProArg-free DFMO / CD and *Dhps* KD / sh-contrl shows no correlation.

f) Western blot analysis of eIF5A hypusination upon treatment with GC7 6.25 uM of GC7 for 5 days of neuroblastoma cell line IMR5 used for ribosome profiling.

g) *GC7* treatment shows minimal changes in ribosome P site occupancy relative to control despite an evident hypusination defect and does not show pausing at adenosine-ending codons.

h) Direct comparison of the translation phenotype in ProArg-free DFMO /EvaCD and GC7 / control treatment shows no correlation.

b-e: Reprocessed and reanalyzed data from Nakanishi *et al*. $n = 2$ per group.

f-h: Data generated as part of this study. $n = 3$ per group.

Abbreviations: CD, control diet; ProArg-free, proline arginine free diet; DFMO, difluoromethylornithine.

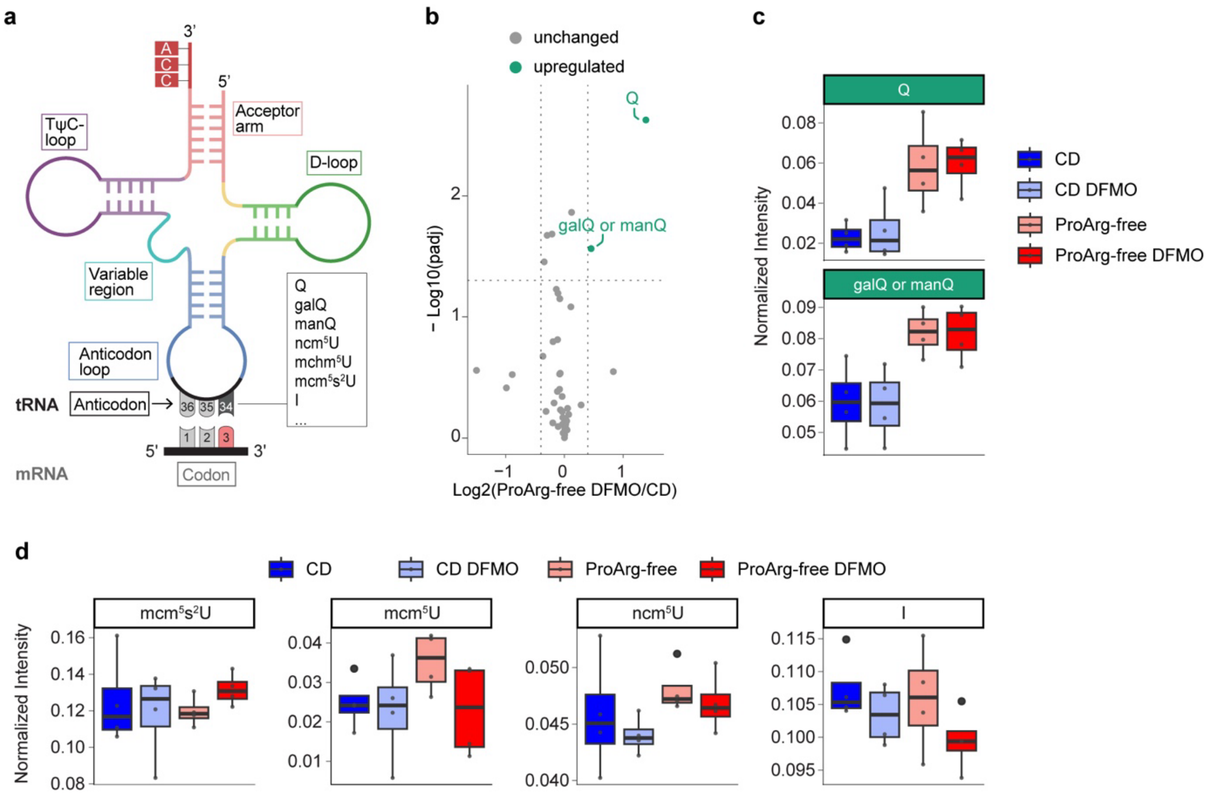

Supplementary Fig. 5: Specific tRNA modifications in neuroblastoma tumors are increased upon dietary intervention

a) tRNA schematic highlighting tRNA modifications at nucleotide 34 (N_{34} detail, box), which fine-tune tRNA anticodon interactions with the nucleotide in position three of the mRNA codon and define ribosome stalling upon polyamine depletion (A-ending, detail).

b) Differential analysis of modified nucleoside levels as quantified by mass spectrometry after digestion of tRNAs extracted from neuroblastoma tumors of the different treatment groups. Volcano plot comparing ProArg-free DFMO and CD, with significant (FDR < 0.05) modifications highlighted in green. Two-tailed t-test.

c) Normalized intensities of significantly upregulated queuosine (Q) modifications and its derivatives (galQ and manQ) in ProArg-free and ProArg-free DFMO TH-*MYCN* tumors.

d) Normalized intensities of other tRNA modifications found at N_{34} that are not significantly changed, but still show stalling upon polyamine depletion in TH-*MYCN* tumors.

c and d: Boxplot where the center line represents the median, the box spans the interquartile range (IQR; 25th to 75th percentiles), and whiskers extend to 1.5IQR. Data points beyond this range are shown as outliers (solid black).

b-d: *n* = 4 per group.

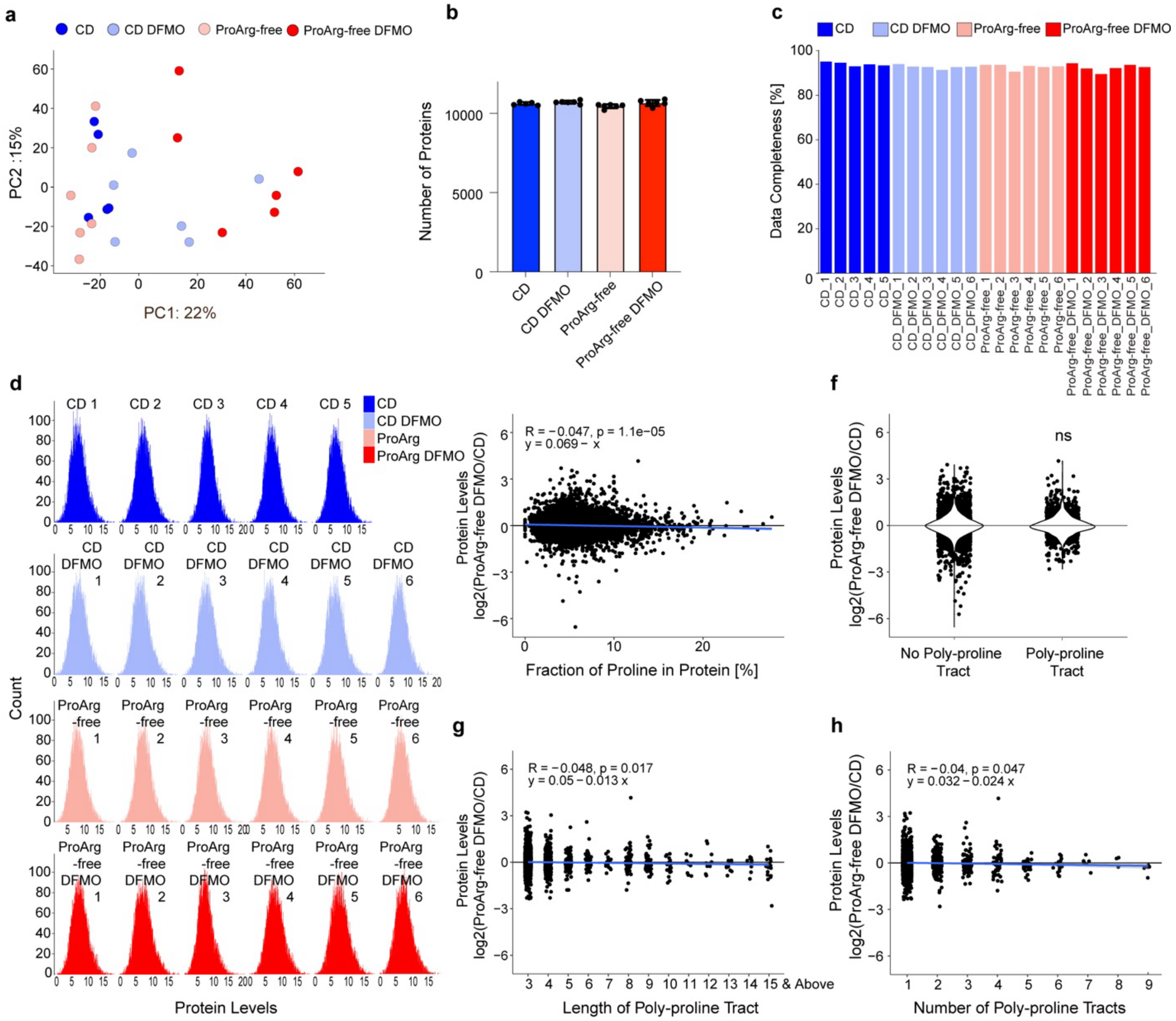

Supplementary Fig. 6: Proteomics quality control and evaluation of protein levels in relation to proline content

a) Global proteomic signatures across all treatment arms analyzed by principal component analysis.

b) Number of proteins measured per treatment group. Mean ± s.e.m..

c) Data completeness of each proteomics analytical measurement, with percentage of proteins captured by each sample per group.

d) Distribution per sample of protein levels.

e) Correlation between relative protein levels in the combined diet-drug treatment (ProArg-free DFMO vs. CD) and the percentage of prolines from all amino acids of the respective protein.

f) Comparison of relative change in protein levels induced by combined diet-drug treatment depending on the presence or absence of at least one poly-proline tract. Poly-proline tracts are defined by having >=3 prolines in a row.

g) Relative protein levels in combined diet-drug treatment grouped by the length of poly-proline tract in the protein.

h) Change in protein levels in the ProArg-free DFMO group depending on the number of poly-proline tracts in the protein.

For a-h: CD DFMO, ProArg-free and ProArg-free DFMO *n* = 6; CD *n* = 5. Abbreviations: CD control diet; ProArg-free proline arginine -free diet; DFMO difluoromethylornithine.

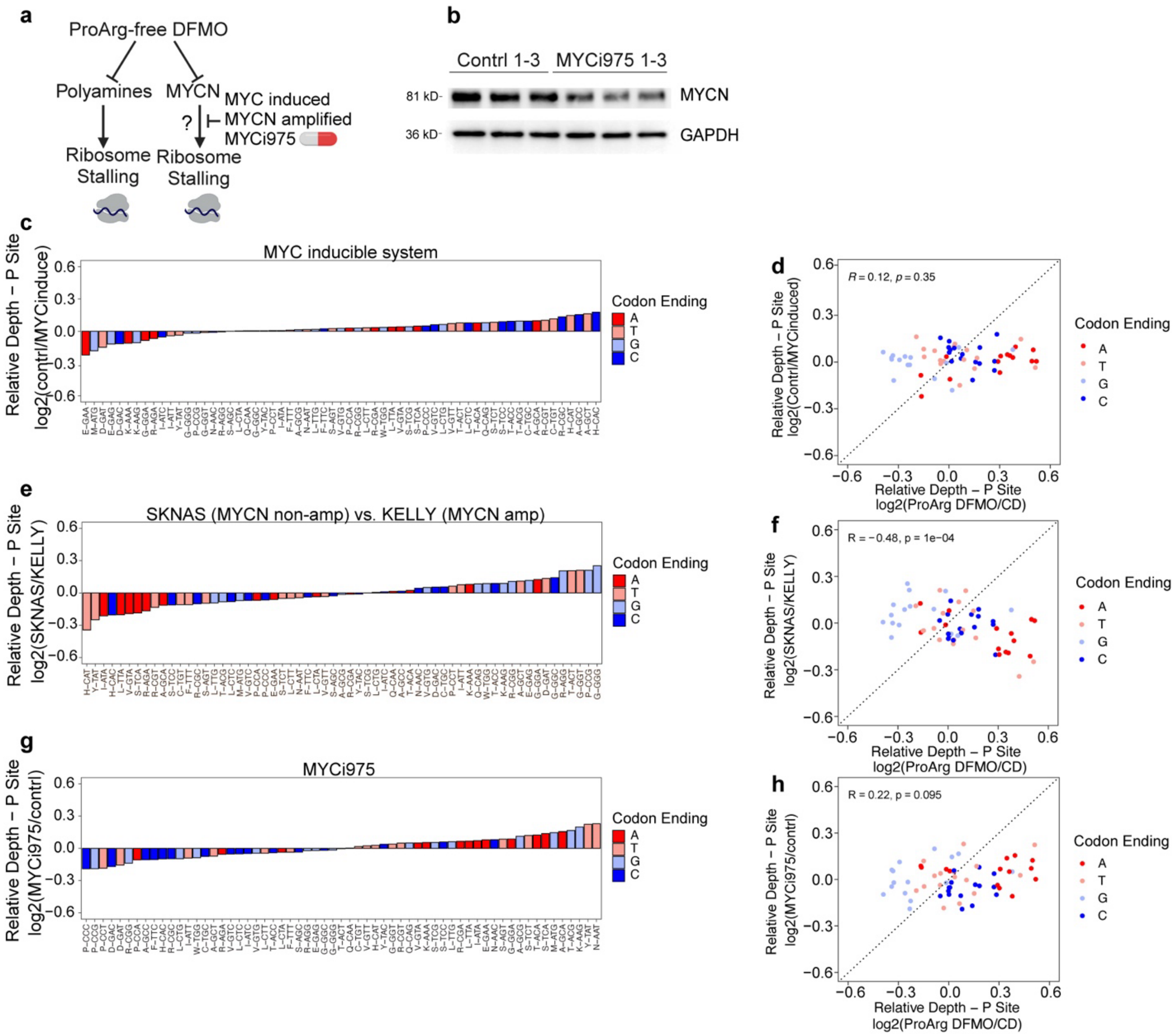

Supplementary Fig. 7: MYC/MYCN inhibition does not cause polyamine depletion codon specific translation defects

a) Background schematic of different approaches to evaluate the role of MYC(N) in driving the translation phenotype of ribosome pausing at adenosine-ending codons by genetic models or pharmacological interventions to modulate MYCN protein abundance.

b) Western blot analysis shows decreased MYCN upon treatment of the neuroblastoma cell line IMR5 with 5 uM MYCi975 for 4 days.

c) Changes in relative ribosome occupancy before and after *MYC* induction in the U2OS cell line. The codon-specific relative read depth (control / *MYC*-induced) at the ribosomal P site does not show pausing at adenosine-ending codons. *n* = 2 per group.

d) Direct comparison of the codon specific translation phenotype in ProArg-free DFMO / CD and the control / MYC induction in U2OS shows no correlation. Both comparisons are low / high MYC. Pearson correlation coefficient.

e) Relative codon-specific ribosome occupancy in neuroblastoma cell lines *MYCN* non-amplified / amplified. Amplification status, and therefore MYCN protein levels, do not impact occupancy of adenosine-ending codons at ribosomal P site. *n* = 3 per group.

f) Direct comparison of the codon-specific translation phenotype in ProArg-free DFMO / CD and the MYCN non-amplified / amplified pair shows no correlation. Pearson correlation coefficient

g) Codon-specific occupancy (ribosomal P site) after modulating MYCN protein levels by introducing MYCi975 treatment at a dose of 5 uM for 4 days. *n* = 3 per group.

h) Direct comparison of the codon-specific translation phenotype upon MYCN degradation by MYCi975 and the ProArg-free DFMO / CD comparison shows a lack of correlation between the two conditions (MYCN low / MYCN high). Pearson correlation coefficient.

Abbreviations: CD, control diet; ProArg-free, proline arginine free diet; DFMO, difluoromethylornithine.

Supplementary Table S1: Patient data characteristics Fig 1a and b

Patient	age_at_diagnosis_days	nbl_differentiation_N3	inss_stage_final	MYCN_Amplification
1	301	2-Poorly differentiated (<5% differentiating neuroblasts)	4-Stage 3	Amplified
2	211	2-Poorly differentiated (<5% differentiating neuroblasts)	1-Stage 1	Not amplified
3	37	2-Poorly differentiated (<5% differentiating neuroblasts)	1-Stage 1	Amplified
4	898	2-Poorly differentiated (<5% differentiating neuroblasts)	5-Stage 4	Amplified
5	786	2-Poorly differentiated (<5% differentiating neuroblasts)	4-Stage 3	Amplified
6	770	2-Poorly differentiated (<5% differentiating neuroblasts)	5-Stage 4	Not amplified
7	813	2-Poorly differentiated (<5% differentiating neuroblasts)	4-Stage 3	Not amplified
8	14	2-Poorly differentiated (<5% differentiating neuroblasts)	1-Stage 1	Not amplified
9	1392	2-Poorly differentiated (<5% differentiating neuroblasts)	5-Stage 4	Amplified
10	83	2-Poorly differentiated (<5% differentiating neuroblasts)	6-Stage 4S	Amplified
11	2057	2-Poorly differentiated (<5% differentiating neuroblasts)	5-Stage 4	Not amplified
12	4206	2-Poorly differentiated (<5% differentiating neuroblasts)	5-Stage 4	Not amplified
13	485	2-Poorly differentiated (<5% differentiating neuroblasts)	4-Stage 3	Not amplified
14	661	2-Poorly differentiated (<5% differentiating neuroblasts)	1-Stage 1	Not amplified
15	1518	2-Poorly differentiated (<5% differentiating neuroblasts)	5-Stage 4	Not amplified
16	855	2-Poorly differentiated (<5% differentiating neuroblasts)	4-Stage 3	Amplified
17	373	2-Poorly differentiated (<5% differentiating neuroblasts)	5-Stage 4	Amplified
18	307	2-Poorly differentiated (<5% differentiating neuroblasts)	5-Stage 4	Amplified
19	519	2-Poorly differentiated (<5% differentiating neuroblasts)	5-Stage 4	Amplified
20	1775	2-Poorly differentiated (<5% differentiating neuroblasts)	1-Stage 1	Not amplified

Supplementary Table S2: Murine diets (control diet and proline arginine free diet) description

Control Diet - Baker Amino Acid Diet (5CC7)	Ingredients (%)	Control Diet - Baker's Amino Acid Diet w/ No Added Proline or Arginine (5WYF)	Ingredients (%)
Corn Starch	41.7824	Corn Starch	41.7824
Sucrose	25.9	Sucrose	25.9
Baker Amino Acid Premix	16	Baker Amino Acid Premix (free of proline and arginine)	16
Baker Amino Acid Mineral Premix	10	Baker Amino Acid Mineral Premix	10
Corn Oil	5	Corn Oil	5
Sodium Bicarbonate	1	Sodium Bicarbonate	1
Baker Amino Acid Vitamin Premix	0.2	Baker Amino Acid Vitamin Premix	0.2
Choline Chloride	0.1	Choline Chloride	0.1
Ethoxyquin (a preservative)	0.0136	Ethoxyquin (a preservative)	0.0136
DL-Alpha Tocopheryl Acetate (Form Vitamin E)	0.004	DL-Alpha Tocopheryl Acetate (Form Vitamin E)	0.004

Supplementary Table S3: Short hairpin (Sh) sequence and primers for qPCR

Target	shRNA Sequence	Genes	Forward Primers(5'-3')	Reverse Primers(5'-3')
shODC1 #1	ACGGGCGAAAGAGCTAAATAT	Human-CENPR	GCGTTTCCTTTGGCGGATTT	AGTGATCTTTTAACAGGCATTCTGA
shODC1 #2	TTTGTGAGAAGCTGGAAATAT	Human-ODC1	GCCATCGTGAAGACCCTTG	GGCAATCCGCAAAACCAACTT
shCENPR #1	GCACAGAAATGGACTATCAAA	Human-KIF2C	CAACTCCAAAATTCCTGCTCC	GAACTGAAAACTGCTTGCGG
shCENPR #2	CGTCATCTTGACAGCTATGAA	Human-MYCN	CTCCGGAGAGGATACCTTGA	TCTCTACGGTGACCACATCG
shKIF2C #3	GCATAAGCTCCTGTGAATATA	Human-GAPDH	TCATGACCACAGTCCATGCC	TCAGCTCTGGGATGACCTTG
shKIF2C #4	GCAGGCTAGCAGACAAATAAG			
shSCR	CAGCAGCAGTTGCTAAAGAAA			
