# Supplementary material for: Reprogramming neuroblastoma by diet-enhanced polyamine depletion
Source: Nature. 2025 Sep 24;646(8085):707–15. doi: 10.1038/s41586-025-09564-0 (PMC12527938; doi:10.1038/s41586-025-09564-0)
Supplement: Supplementary file 2 — Reporting Summary [file 41586_2025_9564_MOESM2_ESM.pdf]

Corresponding author(s): Raphael J. Morscher
Michael D. Hogarty
Joshua D. Rabinowitz

Last updated by author(s): Jul 26, 2025

Reporting Summary

Nature Portfolio wishes to improve the reproducibility of the work that we publish. This form provides structure for consistency and transparency in reporting. For further information on Nature Portfolio policies, see our Editorial Policies and the Editorial Policy Checklist.

Statistics

For all statistical analyses, confirm that the following items are present in the figure legend, table legend, main text, or Methods section.

n/a	Confirmed	
☐	☒	The exact sample size (*n*) for each experimental group/condition, given as a discrete number and unit of measurement
☐	☒	A statement on whether measurements were taken from distinct samples or whether the same sample was measured repeatedly
☐	☒	The statistical test(s) used AND whether they are one- or two-sided<br>*Only common tests should be described solely by name; describe more complex techniques in the Methods section.*
☒	☐	A description of all covariates tested
☐	☒	A description of any assumptions or corrections, such as tests of normality and adjustment for multiple comparisons
☐	☒	A full description of the statistical parameters including central tendency (e.g. means) or other basic estimates (e.g. regression coefficient) AND variation (e.g. standard deviation) or associated estimates of uncertainty (e.g. confidence intervals)
☐	☒	For null hypothesis testing, the test statistic (e.g. *F*, *t*, *r*) with confidence intervals, effect sizes, degrees of freedom and *P* value noted<br>*Give P values as exact values whenever suitable.*
☒	☐	For Bayesian analysis, information on the choice of priors and Markov chain Monte Carlo settings
☒	☐	For hierarchical and complex designs, identification of the appropriate level for tests and full reporting of outcomes
☐	☒	Estimates of effect sizes (e.g. Cohen's *d*, Pearson's *r*), indicating how they were calculated

Our web collection on statistics for biologists contains articles on many of the points above.

Software and code

Policy information about availability of computer code

Data collection	Metabolomics: Xcalibur (Thermofisher.4.4.16.14) was used to collect raw mass spectrometry data, El-Maven (Elucidata, v0.12.1) was used to pick the peaks from the mass spectrometry data and Accucor (R package, v0.3.1) was used to correct for isotope natural abundance. For the polyamine method, Empower3 (Waters, 3.7.0)<br>Proteomics: Spectronaut (Biognosys, v.17.1)<br>Transcriptomics/RiboSeq: Cutadapt (v.3.6.6) and HISAT2 (2.2.1) were used.<br>Deconvolution: CybersortX was ran from website: https://cibersortx.stanford.edu/
Data analysis	R (Statistical Computing, v4.1.0) and Matlab (v.R2019a) were used to analyze the resulting data.

For manuscripts utilizing custom algorithms or software that are central to the research but not yet described in published literature, software must be made available to editors and reviewers. We strongly encourage code deposition in a community repository (e.g. GitHub). See the Nature Portfolio guidelines for submitting code & software for further information.

Data

Policy information about availability of data

All manuscripts must include a data availability statement. This statement should provide the following information, where applicable:

- Accession codes, unique identifiers, or web links for publicly available datasets
- A description of any restrictions on data availability
- For clinical datasets or third party data, please ensure that the statement adheres to our policy

All data displayed in this study is included in the article and its supplementary Information are available as source data.
The RNA-Seq and Ribo-Seq data are accessible under the accession number GEO: GSE244378.
The mass spectrometry proteomics data was deposited to the ProteomeXchange Consortium via the PRIDE partner repository with the dataset identifier: PXD047396.
Any additional data reported in this manuscript is available from the corresponding author upon request.

Research involving human participants, their data, or biological material

Policy information about studies with human participants or human data. See also policy information about sex, gender (identity/presentation), and sexual orientation and race, ethnicity and racism.

Reporting on sex and gender	Biological tumor specimens were derived from male and female study participants.
Reporting on race, ethnicity, or other socially relevant groupings	*Please specify the socially constructed or socially relevant categorization variable(s) used in your manuscript and explain why they were used. Please note that such variables should not be used as proxies for other socially constructed/relevant variables (for example, race or ethnicity should not be used as a proxy for socioeconomic status).*<br>*Provide clear definitions of the relevant terms used, how they were provided (by the participants/respondents, the researchers, or third parties), and the method(s) used to classify people into the different categories (e.g. self-report, census or administrative data, social media data, etc.)*<br>*Please provide details about how you controlled for confounding variables in your analyses.*
Population characteristics	Flash-frozen primary neuroblastoma tumor samples were provided by the Children's Oncology Group (COG) under study number ANBL16B2-Q. International Neuroblastoma Pathology Classification (INPC) histologic parameters. Histology of poorly differentiated neuroblastoma, MYCN amplification status, age, and stage and pathologic classification for every patient was obtained centrally via COG testing and review. Tumor cell content of analyzed samples was confirmed as > 80% percent.
Recruitment	Patients gave consent through the Children's Oncology Group from the NCI.
Ethics oversight	Samples obtained from the COG (Neuroblastoma Biology Committee), request underwent review and approval through a CTEP overseen process: Application ANBL16B2Q, PI, Rabinowitz<br>Committee:<br>Michael D. Hogarty<br>Chair, Neuroblastoma Biology subcommittee<br>Nilsa C. Ramirez<br>Biopathology Center, Medical Director

Note that full information on the approval of the study protocol must also be provided in the manuscript.

Field-specific reporting

Please select the one below that is the best fit for your research. If you are not sure, read the appropriate sections before making your selection.

☒ Life sciences ☐ Behavioural & social sciences ☐ Ecological, evolutionary & environmental sciences

For a reference copy of the document with all sections, see nature.com/documents/nr-reporting-summary-flat.pdf

Life sciences study design

All studies must disclose on these points even when the disclosure is negative.

Sample size	Sample size was based on experimental feasibility, sample availability, and N necessary to obtain definitive results. Exact sample size for each tissue type is listed in figure legend. Each sample is a tissue from a separate mouse (biological replicate).
Data exclusions	Data from mice where the infusion led to no carbon-13 tracer enrichment were discarded, likely interpreted as infusion failed due to catheter failure.
Replication	All experiments conducted were shown in the figures with confirmation using alternative approaches. No further replication completed.
Randomization	Infusions experiment: mice were randomly assigned to different experimental groups.<br>Intervention experiments: original diet studies in the THMYCN mice (where DFMO was started DOL1 and the diet changed later), we

randomized entire litters of THMYCN+/- matings to a treatment arm, and included only the THMYCN+/+ offspring on the trial, per our design All other animal studies used a traditional randomization to treatment assignment.

Blinding

Due to the nature of the treatments necessitating the provision of DFMO in drinking water and therapeutic diets, investigators could not be blinded to the treatment group while completing the murine trials..All had appropriate "placebo" or sham therapies
During analysis, all samples were analyzed together and the same analysis settings were used for all tissues, so tissue identity or timepoint could not govern the quantification of mass spectrometry data.

Reporting for specific materials, systems and methods

We require information from authors about some types of materials, experimental systems and methods used in many studies. Here, indicate whether each material, system or method listed is relevant to your study. If you are not sure if a list item applies to your research, read the appropriate section before selecting a response.

Materials & experimental systems

n/a	Involved in the study
☐	☒ Antibodies
☐	☒ Eukaryotic cell lines
☒	☐ Palaeontology and archaeology
☐	☒ Animals and other organisms
☒	☐ Clinical data
☒	☐ Dual use research of concern
☒	☐ Plants

Methods

n/a	Involved in the study
☒	☐ ChIP-seq
☒	☐ Flow cytometry
☒	☐ MRI-based neuroimaging

Antibodies

Antibodies used

REAGENT (experiment used) Supplier Clone name Lot number Validation
Antibodies
MYCN (Immunoblotting) Santa Cruz sc-53993 B8.4.B A1724 Validated by company for Western blot using whole cell lysates from non-transfected 293T cells (sc-117752) and 293T cells transfected with mouse N-Myc (sc-121906). Further confirmed in our study by MYCN inhibition using MYCi975
EIF5A (Isoelectric Focusing/Immunoblotting) BD Transduction Laboratories 611976 26/eIF-5a 2045903 Validated by company for Western blot analysis using Jurkat cell lysates.
ODC1 (Immunoblotting) Abcam ab270269 ODC1/3636R ND Validated by company for Western blot analysis using PC3 cell lysates. Further confirmed in our study by ODC1 knockdown using shRNA.
Anti-Hypusine Merck Millipore ABS1064 Polyclonal 4105257 Validated by company for Western blot analysis using HEK293 cells transfected with GFP-elF5a wild-type.
CENPR (Immunoblotting) Proteintech 10743-1-AP Polyclonal 00073963 Validated by company for Western blot analysis using K-562 cell lysates. Further confirmed in our study by CENPR knockdown using shRNA.
KIF2C (Immunoblotting) Proteintech 12139-1-AP Polyclonal 00002856 Validated by company for Western blot analysis using various lysates(DU 145, PC-3, mouse testis, rat mouse testis). Further confirmed in our study by KIF2C knockdown using shRNA.
DHPS (Immunoblotting) Proteintech 11184-1-AP Polyclonal 00098704 Validated by company for Western blot analysis using Hela cell lysates. Further confirmed in our study by DHPS knockdown using shRNA.
Anti-Puromycin(Immunoblotting) Merck Millipore MABE343 12D10 4191133 Validated by company for Western blot analysis using HEK293 cell lysates treated with Puromycin and Cyclohexamide, or with Puromycin only.
HRP-GAPDH (Immunoblotting) Proteintech HRP-60004 1E6D9 21010937 Validated by company for Western blot analysis using various lysates(Hela, HEK-293, HepG2, Jurkat, K-562, HSC-T6, NIH/3T3, 4T1, C6).
Acetylated eIF5A (Lys47) (Immunoblotting) Boster Bio P01727 Polyclonal Validated by company for Western blot using L929 cells. Further confirmed in our study by IMR5 protein lysates treated with 500uM DFMO for 5 days and comparing bands with isoelectric blots.
Ki67 (Immunohistochemistry) Abcam ab16667 SP6 Validated by company for immunohistochemistry using Ki67 knockout HeLa cells.
β-Tubulin (Immunoblotting) Cell Signaling Technology 86298S D3U1W 3 Validated by company for Western blot analysis using various lysates (HeLa, NIH/3T3, KNRK cells, rat brain).
HRP-Goat Anti-Mouse Recombinant Secondary Antibody (Immunoblotting) Proteintech RGAM001 20000844
HRP-Goat Anti-Rabbit Recombinant Secondary Antibody (Immunoblotting) Proteintech RGAR001 20000851

Validation

We independently validated the antibodies using neuroblastoma cell lines under increasing concentration of DFMO. Other validations are described bellow with the antibody

Eukaryotic cell lines

Policy information about cell lines and Sex and Gender in Research

Cell line source(s)

Childhood cancer repository https://www.cccells.org/index.php
Bellow are Cellosaurus ID:
SK-N-SH - RRID:CVCL_0531
SMS-SAN - RRID:CVCL_7136
LA-N-5 - RRID:CVCL_0389

CHLA-90 - RRID:CVCL_6610
IMR5 - RRID:CVCL_1306
SKNBE2 - RRID:CVCL_0528
SHSY5Y - RRID:CVCL_0019
SHEP - RRID:CVCL_SHEP
HEK293 - RRID: CVCL_0045

Authentication	Cell lines were authenticated by STR.
Mycoplasma contamination	All cell lines were repeatedly confirmed negative for mycoplasma.
Commonly misidentified lines (See ICLAC register)	No ICLAC commonly misidentified cell lines were used in this study.

Animals and other research organisms

Policy information about studies involving animals; ARRIVE guidelines recommended for reporting animal research, and Sex and Gender in Research

Laboratory animals	Mouse: TH-MYCN. Male and female HOP colony from William A. Weiss, UCSF;<br>Age: TH-MYCN survival study - enrolled at 28 days of life and evaluated for up to 300 days of life. "Late start" study - enrolled at 35-50 days of life and evaluated for 14 days after enrollment. NCr-nu mice - enrolled at 4-6 weeks of life and evaluated for up to 100 days after enrollment.<br>NCr-nu mouse information: Species: Mus musculus, Strain: Crl:NU-Foxn1nu outbred, athymic mice. Obtained at 4-6 weeks of life from Charles River.<br>CD1 nu 6 week old female<br>Animal Husbandry Conditions: (same for both TH-MYCN, NCr-nu mice and CD1 nu) : 12 hours of dark (6PM-6AM). The rodent holding rooms were maintained at a temperature range of 66F - 78F with an ideal setpoint of 72F. The humidity was maintained within a range of 30% - 70% with an ideal setpoint of 50%.
Wild animals	Study did not involve wild animals
Reporting on sex	Both female and male were included and the heterogeneity is reflected.
Field-collected samples	Study did not involve field-collected samples.
Ethics oversight	Animal studies followed protocols approved by the Princeton University and Children's Hospital of Philadelphia Institutional Animal Care and Use Committees.

Note that full information on the approval of the study protocol must also be provided in the manuscript.

Plants

Seed stocks	Study did not involve plants
Novel plant genotypes	*Describe the methods by which all novel plant genotypes were produced. This includes those generated by transgenic approaches, gene editing, chemical/radiation-based mutagenesis and hybridization. For transgenic lines, describe the transformation method, the number of independent lines analyzed and the generation upon which experiments were performed. For gene-edited lines, describe the editor used, the endogenous sequence targeted for editing, the targeting guide RNA sequence (if applicable) and how the editor was applied.*
Authentication	*Describe any authentication procedures for each seed stock used or novel genotype generated. Describe any experiments used to assess the effect of a mutation and, where applicable, how potential secondary effects (e.g. second site T-DNA insertions, mosiacism, off-target gene editing) were examined.*
